# *Arabidopsis* Clade I TGA Factors Regulate Apoplastic Defences against the Bacterial Pathogen *Pseudomonas syringae* through Endoplasmic Reticulum-Based Processes

**DOI:** 10.1371/journal.pone.0077378

**Published:** 2013-09-27

**Authors:** Lipu Wang, Pierre R. Fobert

**Affiliations:** 1 Department of Biology, University of Saskatchewan, Saskatoon, Saskatchewan, Canada; 2 National Research Council Canada, Saskatoon, Saskatchewan, Canada; Instituto de Biología Molecular y Celular de Plantas, Spain

## Abstract

During the plant immune response, large-scale transcriptional reprogramming is modulated by numerous transcription (co) factors. The *Arabidopsis* basic leucine zipper transcription factors TGA1 and TGA4, which comprise the clade I TGA factors, have been shown to positively contribute to disease resistance against virulent strains of the bacterial pathogen *Pseudomonas syringae*. Despite physically interacting with the key immune regulator, NON-EXPRESSOR OF PATHOGENESIS-RELATED GENES 1 (NPR1), following elicitation with salicylic acid (SA), clade I function was shown to be largely independent of NPR1. Unlike mutants in NPR1, *tga1-1 tga4-1* plants do not display reductions in steady-state levels of SA-pathway marker genes following treatment with this phenolic signaling metabolite or after challenge with virulent or avirulent *P. syringae*. By exploiting bacterial strains that have limited capacity to suppress *Arabidopsis* defence responses, the present study demonstrates that *tga1-1 tga4-1* plants are compromised in basal resistance and defective in several apoplastic defence responses, including the oxidative burst of reactive oxygen species, callose deposition, as well as total and apoplastic PATHOGENESIS-RELATED 1 (PR-1) protein accumulation. Furthermore, analysis of *npr1-1* and the *tga1-1 tga4-1 npr1-1* triple mutant indicates that clade I TGA factors act substantially independent of NPR1 in mediating disease resistance against these strains of *P. syringae*. Increased sensitivity to the *N*-glycosylation inhibitor tunicamycin and elevated levels of endoplasmic reticulum (ER) stress marker genes encoding ER-resident chaperones in mutant seedlings suggest that loss of apoplastic defence responses is associated with aberrant protein secretion and implicate clade I TGA factors as positive regulators of one or more ER-related secretion pathways.

## Introduction

Plants employ an active immune system to detect and fight off invading microbial pathogens. The first and most ancient layer of this system relies on the recognition of conserved microbe-associated molecular patterns (MAMPs), such as bacterial flagellin and elongation factor (EF)-Tu, by plant pattern recognition receptors (PRRs) located on the plasma membrane [1,2]. The resulting MAMP-triggered immunity (MTI) is effective at preventing colonization from most microbes. However, a small number of adapted pathogens have successfully evolved mechanisms to suppress MTI and promote disease. For example, the Gram-negative bacterial pathogen *Pseudomonas syringae* delivers effector proteins into the plant cytoplasm through a type III secretion system (T3SS) that collectively interfere with multiple steps of MTI, promoting pathogen growth and the development of disease [3]. The ensuing state is known as effector-triggered susceptibility (ETS). Plants, in turn, have acquired Resistance (R) proteins to detect pathogen effectors or their effects on host targets, thus rendering them avirulence factors and resulting in a strong immune response known as effector-triggered immunity (ETI). ETI is usually accompanied by a hypersensitive cell death response (HR) at the site of infection to limit the access of the pathogen to water and nutrients. A component of MTI and ETI entails the production of mobile signals that leads to systemic acquired resistance (SAR), a broad-range disease resistance against subsequent attacks by otherwise virulent pathogens [4,5]. Signalling associated with all of the above forms of immunity rely on the phenolic metabolite salicylic acid (SA) [6]. In the model plant *Arabidopsis thaliana*, SA signals through the key immune regulator NONEXPRESSOR OF PATHOGENESIS-RELATED GENES 1 (NPR1; At1g64280) [7]. NPR1 and its paralogues, NPR3 (At5g45110) and NPR4 (At4g19660), were recently shown to bind SA and function as SA receptors [8,9].

Most plant pathogenic bacteria colonize tissues intercellularly [10]. Accordingly, plants have evolved active extracellular defence mechanisms. These may be elicited by MAMPs, avirulence factors, or a combination of both depending on the specific host-pathogen interaction. Regardless of which molecule is recognized by the plant, many subsequent events are similar [11]. One well-characterized and very rapid response following recognition of bacterial phytopathogens is a transient apoplastic burst of reactive oxygen species (ROS) [12]. This oxidative burst can function as an antibiotic agent directly or contribute indirectly to defence by causing cell wall cross-linking and acting as a secondary stress signal to induce defence responses [2]. At a later time following pathogen detection, the plant cell wall is reinforced with several polymers in regions of pathogen attack. Most commonly observed is the deposition of papillae containing the β 1,3 glucan callose, lignin-like polymers, phenolics, and structural proteins [13]. In addition to these physical barriers, plant cells secrete toxic cocktails of antimicrobial compounds and pathogenesis-related (PR) proteins in response to pathogen challenge [14].

Defence-related extracellular or membrane proteins must fold into their native conformation and undergo posttranslational modifications in the endoplasmic reticulum (ER) before reaching their final destination (reviewed in 15,16). When protein folding is inhibited or when the folding machinery is overloaded under stressful conditions, unfolded or misfolded proteins may accumulate in the ER, causing ER stress and eventually cell death [15,16]. Subsequently, cells activate the unfolded protein response (UPR), which alleviates ER stress by increasing the capacity for protein folding and degradation or by attenuating translation [18]. A major component of the UPR is the transcriptional upregulation of UPR genes, which are highly induced in response to environmental stress, including pathogen attack [15].

Protein folding in the ER is catalyzed by the lectins calnexin and calreticulin that recognize oligosaccharide side chains on glycoproteins as signals, and facilitated by chaperones, co-chaperones, and protein disulfide isomerases [15]. The importance of the ER during plant immunity has been confirmed by reports showing that mutation of genes involved in ER-based protein folding and secretion impair defence responses and compromise disease resistance [16]. For instance, loss-of-function mutations in several genes involved in ER *N*-glycosylation affect the biogenesis of the PRR EF-Tu receptor (EFR; At5g20480) and impair defence responses induced by elf18, a peptide derived from EF-Tu [19-23]. Mutation of genes encoding ER-resident chaperones, such as BiP2 (BINDING PROTEIN 2; At5g42020) and DAD1 (DEFENDER AGAINST APOPTOTIC DEATH 1; At1g32210), impair SAR against a virulent isolate of *P. syringae* [24,25]. This phenotype is correlated with reduced apoplastic accumulation of the SAR marker protein PR-1 (At2g14610), which is synthesized in the ER [14]. The *bip2* mutant is also hypersensitive to tunicamycin (TM), a potent inhibitor of protein *N*-glycosylation used to trigger ER stress and subsequent UPR [15,26]. Moreover, TM treatment impairs biogenesis and membrane localization of the PRRs EFR and FLAGELLIN SENSING2 (FLS2; At5g46330) [23].

Three transcriptional regulators have been implicated in the control of UPR gene expression in response to biotic stress. The basic region/leucine zipper protein bZIP60 (At1g42990) appears to be functionally homologous to the yeast UPR sensor HAC1 (Homologous to ATF and CREB) [27]. Similar to *HAC1*, *bZIP60* mRNA is subject to an unusual cytoplasmic splicing event involving INOSITOL-REQUIRING ENZYME 1 (IRE1a, At2g17520; IRE1b, At5g24360). Mutation of either bZIP60 or IRE1a compromise SAR and resistance to virulent *P. syringae* [25]. Loss of IRE1a also impairs apoplastic accumulation of PR-1. NPR1 upregulates the expression of numerous genes encoding ER-resident proteins involved in protein folding and secretion in anticipation of PR protein production [24]. NPR1-dependent genes encoding ER proteins are enriched in the *TL1 cis*-element [24] that is recognized by the heat shock factor-like protein TBF1 [28]. Mutation of *TBF1* reduces apoplastic accumulation of PR-1 and impairs both SA- and elf18- induced disease resistance.

The TGA family of bZIP transcription factors has been implicated in the regulation of plant defence responses. Members of clade II (TGA2 [At5g06950], TGA5 [At5g06960], TGA6 [At3g12250]) and clade III (TGA3 [At1g22070] and TGA7 [At1g77920]) bind to the *as-1* element in the promoter of the *PR-1* gene known to be required for expression in response to SA and interact with NPR1 [29-32]. NPR1 stimulates the DNA-binding activity of these transcription factors *in vitro* [30,33] and interacts with TGA2 to form an SA-dependent enhanceosome capable of transactivating *PR-1* [34]. Reverse genetics of clade II TGA factors has established that they have redundant functions and are essential for SA-induced *PR* gene expression and pathogen resistance [35] while the clade III factor TGA3 is required for basal resistance [36] as well as a novel form of cytokinin-induced resistance [37] against virulent *P. syringae*. The TGA factors belonging to clade I (TGA1, [At5g65210] and TGA4, [At5g10030]) do not interact with NPR1 in yeast or non-infected plant cells because of the presence of two oxidized cysteine residues in TGA1 and TGA4 [38]. However, reduction of these cysteines in leaves following SA-treatment enables the interaction with NPR1. *In vitro* S-nitrosylation enhances DNA-binding activity of TGA1 in the presence of NPR1 [39]. Analysis of *Arabidopsis* T-DNA insertion alleles indicated that clade I TGA factors contribute to basal resistance against virulent *P. syringae* [32,36,39] and ETI against an avirulent race of the oomycete *Hyaloperonosporaarabidopsidis* [32]. Epistasis and microarray analyses revealed that a substantial portion of clade I TGA function is independent of NPR1 [32]. Although the *tga1-1 tga4-1* double mutant is more susceptible to virulent pathogens, expression of defence-related transcripts in leaves was not reduced in mutant compared to wild type plants following application of SA. Furthermore, the expression of SA-marker genes, including *PR-1*, *PR-2* (At3g57260) and *PR-5* (At1g75040), were not reduced following challenge with virulent and avirulent *P. syringae* [32].

The present study was undertaken to elucidate the defence responses regulated by clade I TGA factors. The majority of pathological studies of *Arabidopsis* TGA factors to date have relied on host-compatible interactions involving virulent pathogens (i.e. ETS); however, since these represent immune states with compromised MTI and weak ETI [1], they are not ideal for the analysis of defence responses. While the collective host-range of *P. syringae* is very broad, specific strains generally cause disease on one or a small number of plant species, even a few cultivars of a single plant species [40]. To this end, *P. syringae* isolates having limited capacity to suppress *Arabidopsis* defence responses were exploited in the present study. Results obtained indicate that clade I TGA factors are positive regulators of MTI, acting mostly independent of NPR1. Clade I TGA factors contribute to well-known apoplastic defences including the oxidative burst, callose deposition and PR-1 secretion. The *tga1-1 tga4-1* mutant is also more sensitive to TM, suggesting a role for these TGA factors in regulating ER stress and the UPR.

## Materials and Methods

### Plant material, growth conditions and pathogen infection

The *Arabidopsis thaliana tga1-1, tga4-1*, and *npr1-1* single mutant, *tga1-1 tga4-1* double mutant, and *tga1-1 tga4-1 npr1-1* triple mutant, all in ecotype Columbia (Col-0) were previously described [32,41]. Seed surface sterilization and plant growth conditions were as per [32]. *Pseudomonas syringae* strains *Pst hrcC* ^*-*^ and *Psp* 1448a were propagated at 28°C on King’s *B* *medium* (Becton Dickinson, http://www.bdbiosciences.com) containing Rifampicin (100 mg l^-1^). Infection of plants and quantification of pathogen growth were performed according to [32] with *Pst hrcC* ^*-*^ at 10^5^ colony forming units (cfu) ml^-1^ or *Psp* 1448a at 10^6^ cfu ml^-1^. Log-transformed data were analyzed statistically using Analysis of Variance (ANOVA), General Linear Model (SAS Institute Inc., http://www.sas.com) and significance was determined by post-hoc test (Tukey’s Honest Significant Difference test) [42].

### Callose deposition

Four-week-old leaves were infiltrated with 10^8^ cfu ml^-1^ of *Pst hrcC* ^*-*^ or *Psp* 1448a, 5 µM flg22 or 10 mM MgCl_2_. After 12 h, whole leaves were harvested, stained with 0.01% Aniline blue [43], and observed with a Leica FluoIII (Leica Microsystems, http://www.leica-microsystems.com) epifluorescence microscope. The numbers of callose depositions were counted using the GENETOOLS software (Syngene, http://www.syngene.com) and verified by manual counts. Three plants for each genotype were analyzed. Four leaves were collected from each plant and four areas on each leaf were photographed for counting callose deposits.

### ROS measurement

ROS production from leaf tissue was measured by H_2_O_2_-dependent luminescence of luminol [44]. Leaf discs (5 mm diameter; three per well) from four-week-old plants were floated on water overnight before addition of 2 µM flg22 in 200 µl buffer containing 400 µM luminol (Sigma, http://www.sigma.com) and 20 µg ml^-1^ horseradish peroxidase (Sigma). Luminescence was measured using a VICTOR^3^ multilabel spectrometer (PerkinElmer, http://www.perkinelmer.com) for 20 min after the addition of the test solution.

### Kinetic reverse-transcriptase polymerase chain reaction analysis

Total RNA extraction, cDNA production and RT-qPCR were performed as described [32]. Oligonucleotide primers employed are listed in Table S1. Statistical significance between genotypes at the same time point was determined by Student’s *t*-test.

### Protein extraction and western blot analysis

Four-week-old leaves were syringe-infiltrated with bacterial suspensions of *Pst hrcC* ^*-*^ or *Psp* 1448a at a high concentration (10^8^ cfu ml^-1^). Intercellular washing fluids (IWFs) were isolated from leaves as described by 24. Total protein was extracted according to [43]. Protein concentration was determined using the Bio-Rad protein assay (Bio-Rad, http://www.bio-rad.com). Fifty µg. IWFs or total protein were run on 16% Tricine-SDS-PAGE gels [45], transferred to PVDF (polyvinylidene difluoride) membrane (Bio-Rad), and probed with antibodies specific to the PR-1 protein. Two different PR-1 antisera were used and kindly provided by Dr. Daniel J. Kliebenstein (University of California, Davis, CA) [46] and Dr. Darrell Desveaux (University of Toronto, Toronto, ON) (unpublished), respectively. The blots were developed with an enhanced chemiluminescence detection system, according to the manufacturer’s instructions (Millipore, http://www.millipore.com). The same gels were stained with Coomassie Brilliant Blue R250 (Sigma) as a loading control.

### Tunicamycin assays

Surface sterilized seeds were placed on ½ strength Murashige and Skoog (MS) medium containing tunicamycin (TM) (Sigma) at different concentration. At 5 days after sowing, seedlings were transferred to TM-free MS medium and grown for another five days. To quantify growth, seeds were placed on ½ MS and 1% sucrose medium without TM. Five days after sowing, seedlings were immersed in ½ MS liquid with or without 0.8 µg ml^-1^ TM for 6 h. After treatment, seedlings were rinsed three times with TM-free ½ MS liquid, and grown for a further 5 days on TM-free ½ MS agar. Fresh weight of seedlings was measured. For RT-qPCR, 10-day-old seedlings were immersed with 5 µg ml^-1^ TM for the indicated time periods. Four independent batches of seedlings for each condition were used as the source of RNA.

## Results

### Clade I TGA factors contribute to MTI against *P. syringae*

To ascertain the possible role of clade I TGA factors in MTI, *tga* mutants were challenged with two isolates of *P. syringae* previously reported to elicit MTI in the Columbia-0 (Col-0) ecotype of *Arabidopsis thaliana*. The *hrcC* mutant of *P. syringae* pathovar (pv.) *tomato* DC3000 (*Pst hrcC* ^*-*^) does not produce a functional T3SS, and accordingly, is incapable of delivering T3SS-dependent effectors (T3SEs) into the plant cell [47]. In the absence of T3SEs, MTI is the predominant immune response limiting bacterial growth and disease symptoms of Col-0 against *Pst hrcC* ^*-*^ [47]. Isolates of *P. syringae* pv. *phaseolicola* (*Psp*) do not cause disease on *Arabidopsis* . These interactions are classified as type 1 non-host resistance (NHR) and occur in the absence of an HR [48]. Non-host resistance of *Psp* strain 1448a in *Arabidopsis* is largely determined by MTI triggered by the recognition of a conserved 22 amino acid peptide (flg22) derived from bacterial flagellin [49].

**Figure 1:**
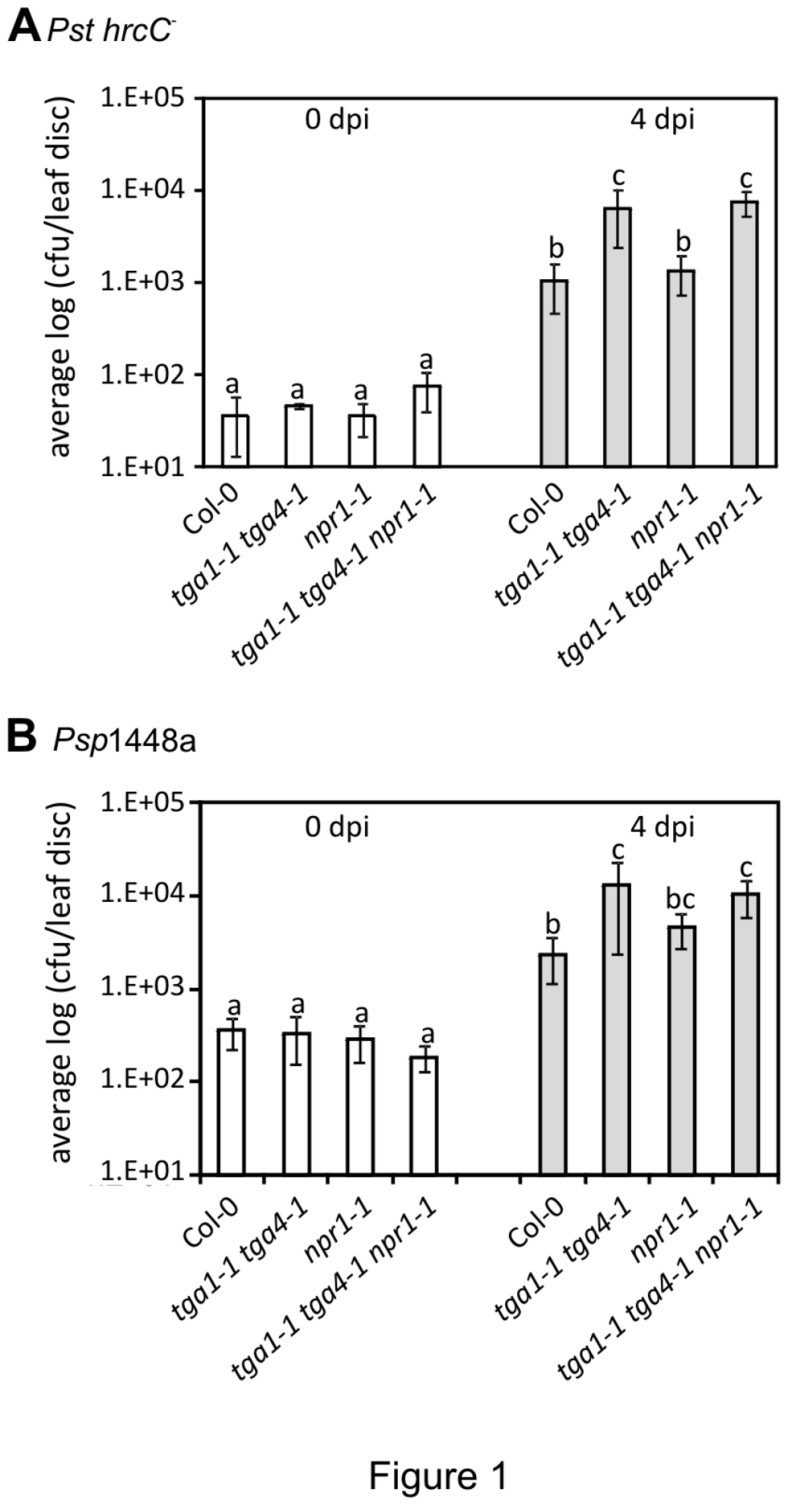
Growth of *Pst hrcC* ^*-*^ (A) and *Psp* 1448a (B) in Col-0, *tga1-1 tga4-1, npr1-1 and tga1-1 tga4-1 npr1-1* plants. Four-week old leaves were syringe-infiltrated with a bacterial suspension of *Pst* *hrcC* ^*-*^ at 10^5^ colony forming units (cfu) ml^-1^ or *Psp* 1448a at 10^6^ cfu ml^-1^. The error bars represent the standard deviation of six replicates, each containing 8 leaf discs from one plant. An ANOVA of the log-transformed data was performed at α = 0.05; treatments with common letters over bars are not significantly different from each other. Post-hoc tests are presented in Table S2. Each experiment was repeated twice with similar results.

Bacterial titres of *Pst hrcC* ^*-*^ or *Psp* 1448a in leaves of four-week-old *tga1-1 tga4-1* and Col-0 plants were similar on the day of infiltration (0 dpi) (Figure 1A, 1B). Four days after infection (4 dpi), leaves of *tga1-1 tga4-1* plants harbored significantly higher titres of both *Pst hrcC* ^*-*^ and *Psp* 1448a than Col-0 (Figure 1A, 1B). To resolve the contribution of individual clade I TGA factors, single mutants were also analyzed. Mutation of neither *TGA1* nor *TGA4* individually significantly affected growth of *Pst hrcC* ^*-*^ or *Psp* 1448a (Figure S1). Significant differences of bacterial titres were only observed when both genes were disrupted in the double mutant (Figure S1). These results indicate that *TGA1* and *TGA4* contribute to resistance against both isolates of *P. syringae* most likely through MTI, and that clade I TGA factors act redundantly.

### Mutation in NPR1 does not affect MTI against *P. syringae*

To determine the relationship between clade I TGA factors and NPR1 towards defence against *Pst hrcC* ^*-*^, this strain was also infiltrated into leaves of *npr1-1* and the *tga1-1 tga4-1 npr1-1* triple mutant. Unlike the *tga1-1 tga4-1* mutant, *npr1-1* did not support higher titres of *Pst hrcC* ^*-*^ at 4 dpi (Figure 1A). Furthermore, *Pst hrcC* ^*-*^ multiplied to similar levels in leaves of the triple *tga1-1 tga4-1 npr1-1* and the double *tga1-1 tga4-1* mutants.

Previous studies demonstrated that NPR1 plays a limited role in NHR against *Psp* [50,51]. Consistent with these studies, bacterial growth in the *npr1-1* mutant at 4 dpi was not statistically different than the wild type (Figure 1B). While the *tga1-1 tga4-1 npr1-1* triple mutant supported significantly higher bacterial titres than wild type, values were not statistically different than the *tga1-1 tga4-1* double mutant (Figure 1B), suggesting that the higher bacterial titres observed in the triple mutant is primarily due to loss of function in clade I TGA factors. Together these results indicate that loss of function in NPR1 does not affect MTI-mediated disease resistance and that clade I TGA factors act mainly in an NPR1-independent fashion with respect to resistance against *Pst hrcC* ^*-*^ and *Psp*.

### The *tga1-1 tga4-1* mutant is not compromised in expression of defence marker genes

Bacterial growth results indicate that MTI-mediated disease resistance mainly requires clade I TGA factors but not NPR1. To further determine what defence responses may be compromised in *tga* mutants, leading to higher bacterial titres, several well-known defence response events associated with MTI were examined in *tga1-1 tga4-1* double mutant. First, to investigate the role of clade I TGA factors in regulating the expression of defence genes during MTI, transcript levels of some well-known SA and MTI markers were quantified in leaves of Col-0 and *tga1-1 tga4-1* after challenge with *Pst hrcC* ^*-*^ or *Psp* 1448a by reverse-transcriptase quantitative PCR (RT-qPCR). In addition to *PR-1* as a marker for SA-dependent genes, the following genes were analyzed. Phenylalanine ammonia lyase 1 (PAL1; At2g37040) is a key enzyme of phenylpropanoid biosynthesis and is involved in lignification during cell wall fortifications at the inoculation site [52]. Its transcripts are rapidly upregulated by *P. syringae hrp* mutants, avirulent *Pst*, and non-host bacteria, but suppressed by virulent *Pst* [53]. FLG22-INDUCED RECEPTOR-LIKE KINASE 1 (FRK1; At2g19190) is a MAMP-induced marker gene [54]. *NONHOST* RESISTANCE *1* (*NHO1*; At1g80460) encodes a glycerol kinase which is required for NHR against *Psp* and a marker for NHR [55,56].

**Figure 2:**
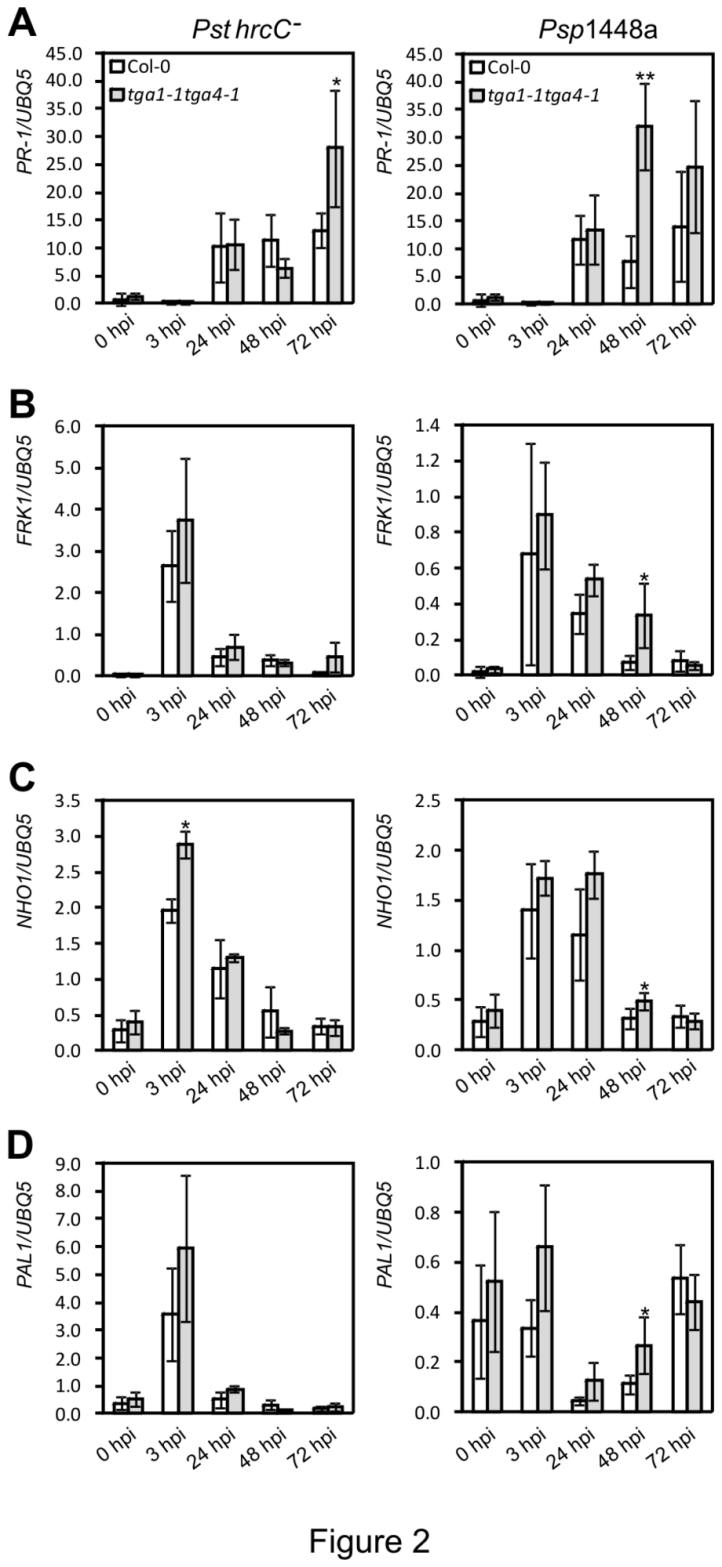
Defence-related gene expression in Col-0 and *tga1-1 tga4-1.* Four-week-old leaves were syringe-infiltrated with 10^8^ cfu ml^-1^ of *Pst* *hrcC* ^*-*^ or *Psp* 1448a. Leaf tissues from three plants were collected and pooled as one sample for RNA isolation. Values were normalized to the expression of *UBIQUITIN5*. An asterisk indicates a statistically significant difference compared with Col-0 at the same time point (p<0.05, Student’s *t*-test), and two asterisks indicate p<0.01. The error bars represent the standard deviation of three biological samples.

Levels of *PR-1* transcripts were substantially higher at 24 h post-infection (hpi) and afterwards than at earlier time points (Figure 2A). When comparing levels in leaves of *tga1-1 tga4-1* and Col-0, no clear trend was observed following challenge with *Pst hrcC* ^*-*^. Levels were lower in the mutant at 48 hpi, but statistically higher in the mutant by 72 hpi (Figure 2A). In contrast, levels of *PR-1* were statistically higher in *tga1-1 tga4-1* at 48 and 72 hpi with *Psp* 1448a (Figure 2A). Transcript levels for the remaining marker genes peaked at 3 hpi and declined thereafter, with the exception of NHO1 following challenge with *Psp*, which declined after 24 hpi (Figure 2B-D). At their peak, levels of transcripts measured in leaves of the *tga1-1 tga4-1* mutant were comparable to those found in Col-0 or higher. These results indicate that clade I TGA factors are not required for the pathogen-induced expression of the marker genes investigated.

### The *tga1-1 tga4-1* mutant is impaired in pathogen- and MAMP-induced callose deposition

**Figure 3:**
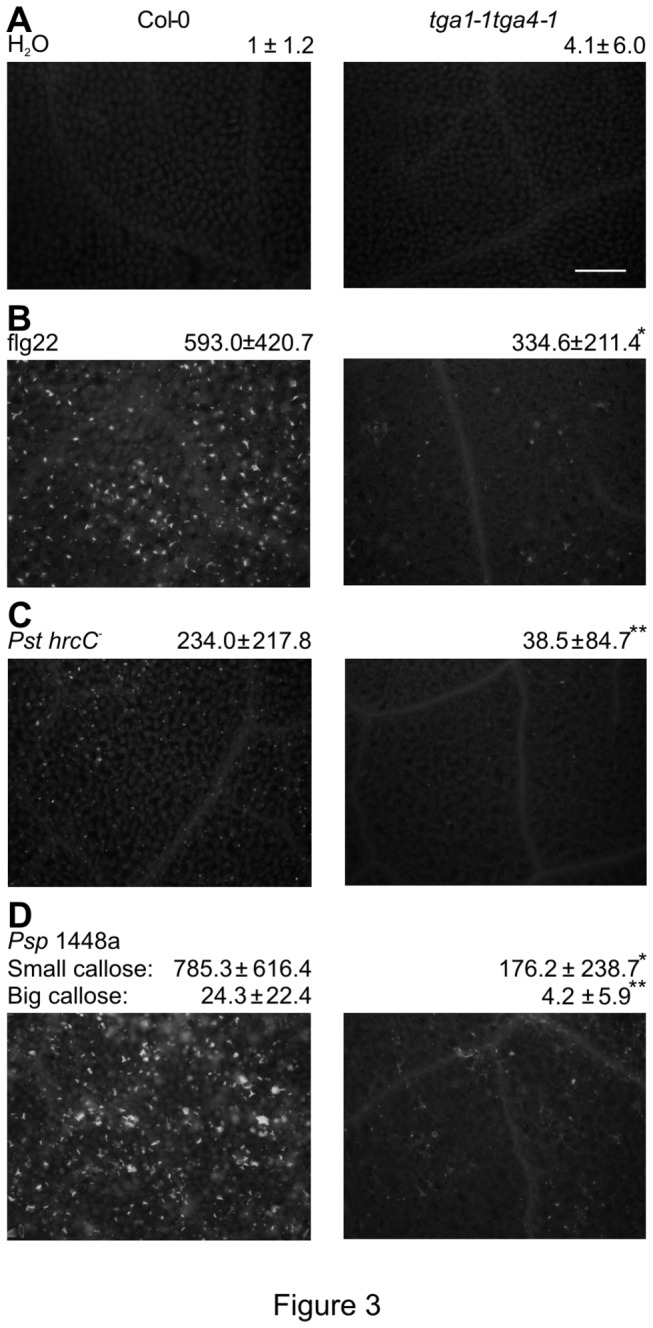
MAMPs- and pathogen-induced callose deposition in Col-0 and *tga1-1 tga4-1* plants. Four-week-old leaves were syringe-infiltrated with 10^8^ cfu ml^-1^ of *Pst* *hrcC* ^*-*^ or *Psp* 1448a, 5µM flg22, and 10mM MgCl_2_ as control. Leaves were stained with Aniline blue and observed under a florescent microscope 12 h after treatment. Microscopic photographs of callose deposits are shown with the number of callose deposits indicated below each. Results are presented as means ± standard deviation. An asterisk indicates a statistically significant difference between Col-0 and *tga1-1* *tga4-1* (p<0.05, Student’s *t*-test), and two asterisks indicate p<0.01. Each experiment was repeated three times with similar results. Scale bar = 0.1mm, all photos are at the same magnification.

Callose deposition was measured as an example of a typical cell wall-associated defence response induced by MAMPs or non-infectious pathogens [57]. The *tga1-1 tga4-1* mutant and Col-0 plants were challenged with *Pst hrcC* ^*-*^ or *Psp* 1448a as described above. To directly monitor the response of clade I TGA mutants to MAMPs, we also treated plants with a purified MAMP, the flg22 peptide [2]. The number and size of callose deposits were measured after staining with Aniline blue. A large number of callose deposits were observed after flg22 treatment and *Pst hrcC* ^*-*^ challenge in leaves of Col-0 plants (Figure 3B and 3C). In leaves of *tga1-1 tga4-1*, the number of callose deposits observed was significantly reduced following flg22 treatment (50%) and *Pst hrcC* ^*-*^ challenge (80%). These results indicate that clade I TGA factors are required for flg22- and *Pst hrcC* ^*-*^- induced callose deposition.

The non-adapted pathogen *Psp* NPS3121 induces two morphologically different types of callose deposits (small and big) in Col-0 [51]. These two types of callose are triggered by MAMPs and T3SEs of *Psp*, respectively. As shown in Figure 3D, infiltration with *Psp* 1448a also triggered both types of callose deposits. A lower number of both big and small callose deposits were induced in the *tga1-1 tga4-1* mutant, indicating that clade I TGA factors are required for both MTI- and ETI-mediated callose deposition.

To examine whether reduction of callose deposition observed in *tga1-1 tga4-1* following challenge with *Pst hrcC* ^*-*^ was associated with reduced transcripts of callose synthase genes in the double mutant, transcript levels of *CALLOSE SYNTHASE 12* (*CalS12*; At4g03550, also known as *POWDERY MILDEW RESISTANCE 4, PMR4*), were quantified. *CalS12* is required for callose deposition in response to fungal and bacterial pathogens [43,58,59] and its transcripts are highly induced by SA and pathogens [60]. Steady-state levels of *CalS12* transcripts in leaves of *tga1-1 tga4-1* plants were slightly lower than those in Col-0 at peak expression (3 hpi) but overall patterns were very similar in the two genotypes (Figure S2). Thus, lost of callose deposition is not associated with reductions in steady-state levels of the key biosynthetic callose synthase gene.

### The *tga1-1 tga4-1* mutant is impaired on MAMP-induced oxidative burst

**Figure 4:**
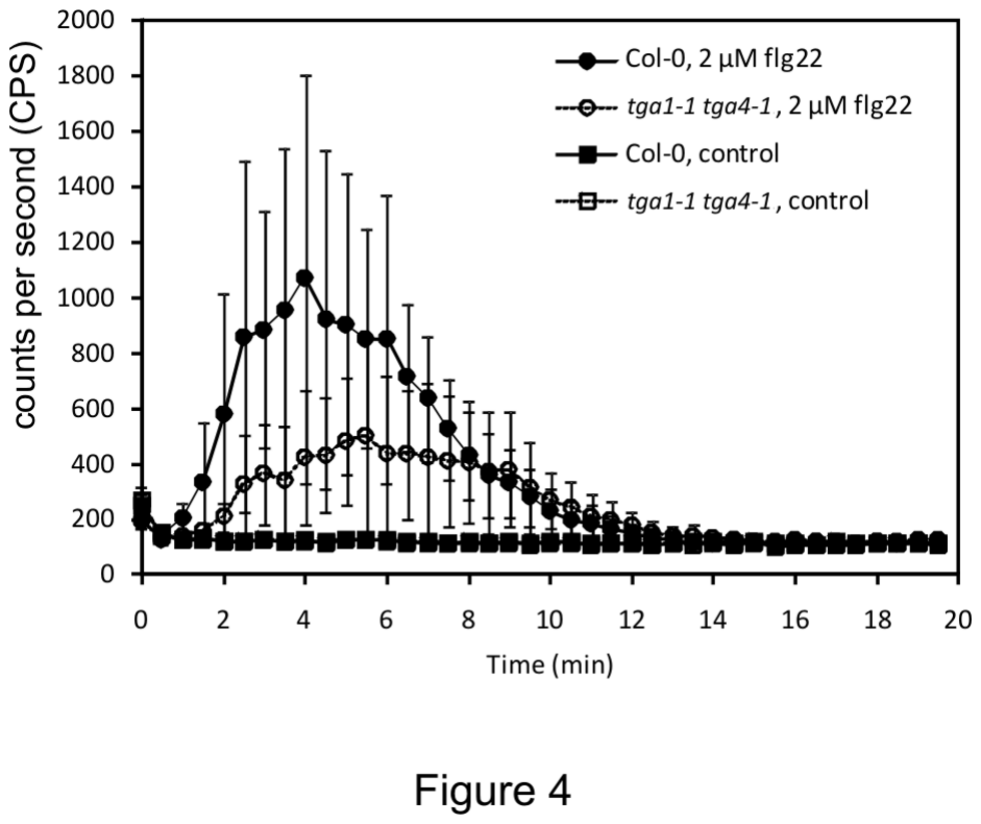
MAMPs-induced oxidative burst in Col-0 and *tga1-1tga4-1* plants. Four-week-old leaf discs (3 per each sample) were treated with or without 2 µM flg22 in the presence of luminol, and the H_2_O_2_ generated was measured every 30 sec after treatment for 20 min. The error bars represent the standard deviation of six replicates. The experiment was repeated five times with similar results.

An oxidative burst is an early defence response triggered upon pathogen perception [2]. To ascertain whether clade I TGA factors are required for MAMP-induced ROS production, levels of hydrogen peroxide (H_2_O_2_) were measured in leaves of the *tga1-1 tga4-1* mutant after treatment with flg22. An oxidative burst was rapidly induced in Col-0 plants, peaking after 4 min (Figure 4). This response was clearly reduced in the double mutant, reaching only about half the intensity of Col-0, indicating that clade I TGA factors are positive regulators of the oxidative burst during MTI.

### Clade I TGA factors are required for pathogen-induced PR-1 protein accumulation

**Figure 5:**
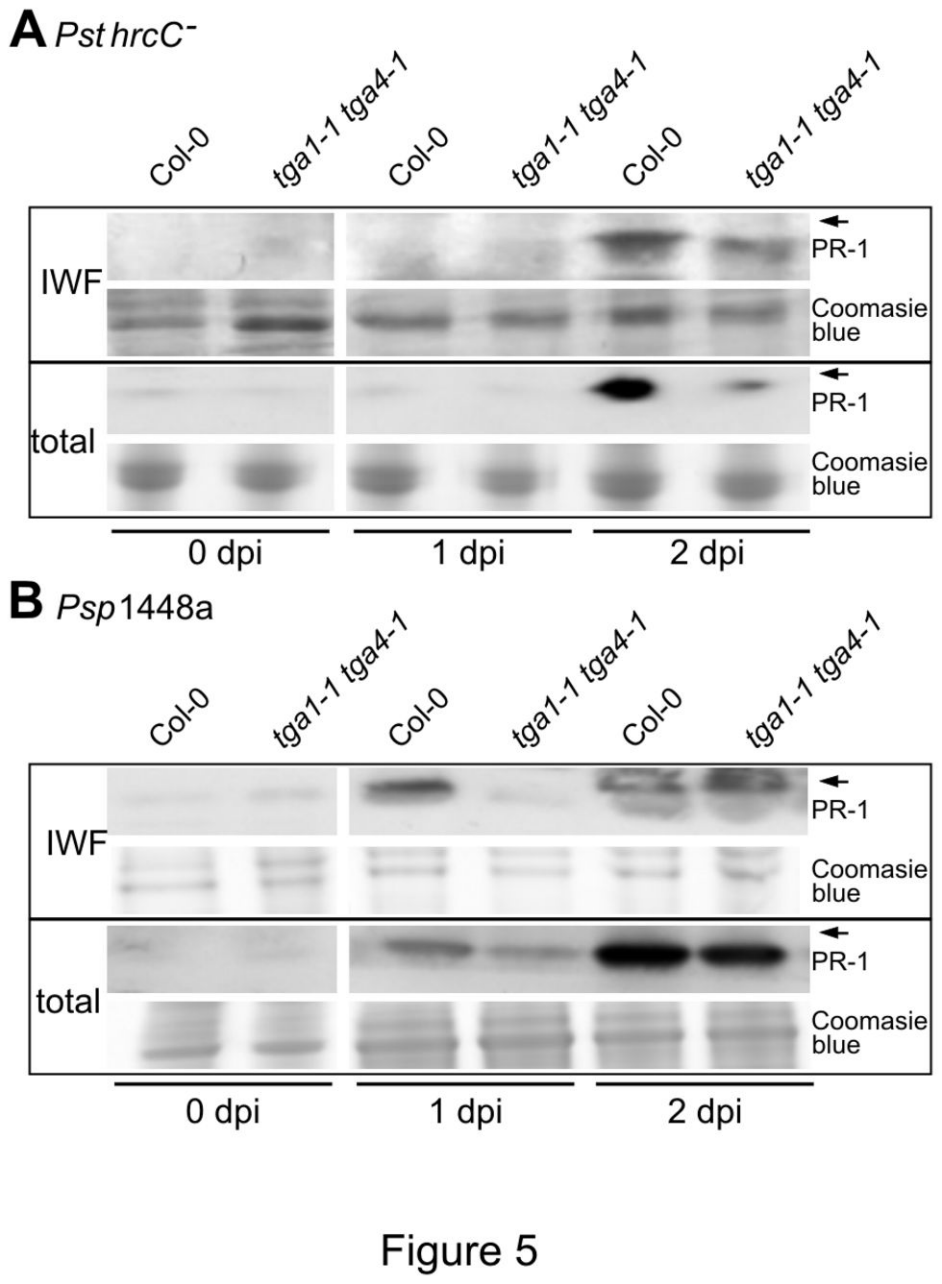
PR-1 protein accumulation in Col-0 and *tga1-1 tga4-1* after pathogen inoculation. Four-week-old leaves were syringe-infiltrated with 10^8^ cfu ml^-1^ of *Pst* *hrcC* ^*-*^ or *Psp* 1448a. Intercellular washing fluids (IWFs) and total protein were collected at 0, 1 and 2 dpi, separated on 16% Tricine-SDS-polyacrylamide gels and blotted with a PR-1 antibody. The *Arabidopsis* PR-1 protein has apredicted molecular weight of 16 kilodaltons (kDa) [74]. Arrows indicate the position of a 17-kDa molecular weight masker. The same gels were stained with Coomassie Brilliant Blue R250 (Sigma) as a loading control. These experiments were repeated three times with similar results.

Another important plant defence response against intercellular bacterial pathogens is the production of extracellular proteins that reinforce cell walls or have antimicrobial activities [10]. To study defence-related production of extracellular proteins, apoplastic accumulation of PR-1 was monitored by immunoblotting of intercellular washing fluids (IWFs) from leaves following challenge with *Pst hrcC* ^*-*^ or *Psp* 1448a (Figure 5). In response to *Pst hrcC* ^*-*^, PR-1 protein was detectable in IWFs from both *tga1-1 tga4-1* and Col-0 leaves at 2 dpi, with mutant leaves accumulating lower levels (Figure 5A and Figure S6). PR-1 was detectable earlier (1 dpi) in IWFs of Col-0 following challenge with *Psp* 1448a (Figure 5B); however, PR-1 was not detected until 2 dpi in IWFs from leaves of *tga1-1 tga4-1* plants. At this time point, levels in the mutant and wild type were comparable. The pattern of total PR-1 accumulation was similar to that observed in IWFs, with lower levels detected in the mutant at 2 dpi following challenge with *Pst hrcC* ^*-*^, and at 1 dpi after infiltration with *Psp* 1448a (Figure 5). Thus, although loss of clade I TGA factors does not impair *PR-1* transcript levels (Figure 2A), it leads to reductions in corresponding protein production.

### The unfolded protein response is impaired in the *tga1-1 tga4-1* mutant

The observation that loss of clade I TGA factors affected callose deposition (Figure 3) and extracellular PR-1 accumulation (Figure 5), but not the steady-state levels of either callose synthase (Figure S2) or *PR-1* transcripts (Figure 2), suggested that the mutant may be affected in some aspect of protein secretion. Examination of genes differentially expressed between leaves of Col-0 and *tga1-1 tga4-1* identified by [32] revealed an enrichment for gene ontology classifications related to the ER, other membranes, extracellular, and protein binding. Of note, several genes known to be involved in ER-based protein folding are up-regulated in *tga1-1 tga4-1* plants (Figure S3). Increased expression of these genes and their products can be a sign of ER stress [17] and suggests that *tga1-1 tga4-1* plants may be constitutively under ER stress and trigger the UPR.

**Figure 6:**
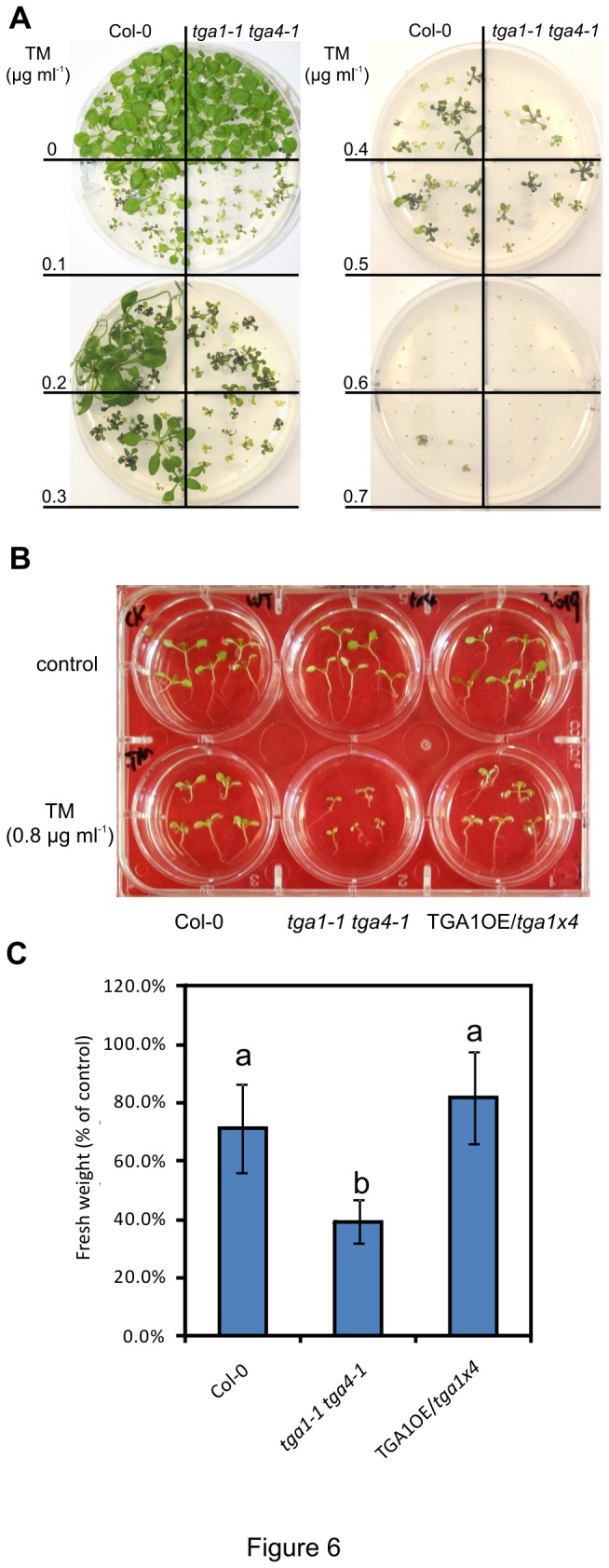
Tunicamycin sensitivity of Col-0 and the *tga1-1 tga4-1* plants. **A**, Five-day-old seedlings of Col-0 and the *tga1-1* *tga4-1* double mutant grown on Murashige and Skoog (MS) agar medium with different concentrations of TM were transplanted to TM-free MS agar and grown for a further 5 days prior to photography. This experiment was repeated three times with similar results. **B**, Five-day-old seedlings of Col-0, *tga1-1* *tga4-1*, and a line overexpressing TGA1 in the *tga1-1* *tga4-1* background (TGA1OE/*tga1x4*) grown on TM-free MS agar were submerged in MS liquid medium with or without 0.8 µg ml^-1^ TM for 6 h, and were allowed to recover for 5 days without TM prior to photography. **C**, Fresh weight of seedlings in (**B**) was quantified. The fresh weight of TM-treated seedlings was divided by the average fresh weight of 5 untreated seedlings to generate percentage of control. The results are averages ± standard deviation (n=5). An ANOVA of data was performed at α = 0.05; treatments with common letters over the error bars are not significantly different from each other. This experiment was repeated twice with similar results.

To test the potential involvement of clade I TGA factors in regulating ER stress, seeds of Col-0 and *tga1-1 tga4-1* were germinated on plates containing different concentrations of TM, an inhibitor of *N*-linked glycosylation that can trigger ER stress [26]. Five days after treatment, seedlings were transferred to TM-free medium for 10 days. In control seedlings transferred from medium lacking TM, no difference in growth between Col-0 and *tga1-1 tga4-1* was observed, indicating that loss of clade I TGA factors does not affect seedling growth (Figure 6A). However, seedlings transferred from media containing different concentrations of TM displayed substantial differences between genotypes, with *tga1-1 tga4-1* seedlings being more sensitive to growth inhibition by TM than Col-0 (Figure 6A).

To quantify the effects of TM on growth, five-day-old seedlings cultured on TM-free solid medium were submerged in liquid media with or without TM for 6 h prior to recovery for 5 days in the absence of TM. The fresh weight of TM-treated seedlings was measured and normalized by the fresh weight of untreated seedlings. Compared to the untreated controls, the fresh weight of Col-0 seedlings was reduced by 30% after TM treatment, indicating that TM effectively inhibited the seedling growth in this assay (Figure 6B). The reduction of fresh weight in *tga1-1 tga4-1* seedlings (60% of non-treated) was statistically lower than measured in Col-0. A transgenic line overexpressing TGA1 in the *tga1-1 tga4-1* background (TGA1OE/*tga1x4*) displayed a similar reduction of fresh weight than Col-0, demonstrating that the differences observed in the mutant was specific to the loss of clade I TGA function.

**Figure 7:**
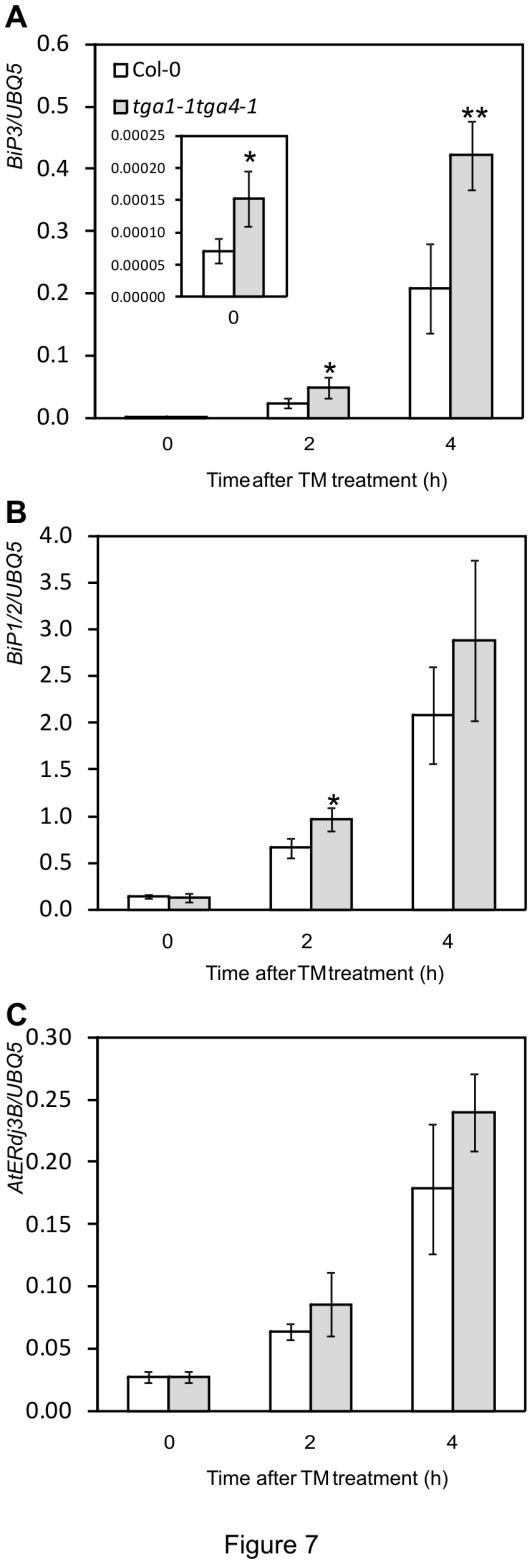
ER stress gene expression in Col-0 and the *tga1-1 tga4-1* plants. Ten-day-old seedlings were immersed with 5 µg ml^-1^ TM for the indicated time periods. Fifty mg samples were collected and pooled for RNA isolation. Values were normalized to the expression of *UBIQUITIN5*. An asterisk indicates a statistically significant difference compared with Col-0 at the same time point (p<0.05, Student’s *t*-test), and two asterisks indicate p<0.01. The error bars represent the standard deviation of four biological samples. The oligonucleotides used in (**B**) did not distinguish between *BiP1* and *BiP2*. Accordlingly, the target is referred to as *BiP1/2*.

To further explore the role of clade I TGA factors in regulating ER stress, the expression pattern of genes encoding ER-resident chaperones was analyzed in *tga1-1 tga4-1* seedling following TM treatment. As shown in Figure 7, TM treatment triggers the rapid accumulation of transcripts for *BiP*_*1/2*_, *BiP3*, and *ERdjB3* in both Col-0 and *tga1-1 tga4-1* seedlings. Levels of these genes in *tga1-1 tga4-1* seedlings were higher than measured in Col-0 (Figure 7). For the *BiP3* gene, transcript levels in *tga1-1 tga4-1* seedlings were also significantly higher than Col-0 before TM treatment (Figure 7A). These results implicate clade I TGA factors in the proper functioning of the ER secretion pathway and indicate that increased susceptibility of *tga1-1 tga4-1* plants to TM is not caused by the loss of ER chaperone gene expression.

## Discussion

Through the analysis of the *tga1-1 tga4-1* double mutant, this study demonstrates the involvement of clade I TGA factors in apoplast-based defence responses against microbial pathogens. Specifically, the mutant is defective in well-characterized inducible responses, including the production of ROS (Figure 4), the deposition of callose in papillae (Figure 3) and the accumulation of PR-1 protein (Figure 5). Given the increased sensitivity of *tga1-1 tga4-1* seedlings to the UPR trigger TM (Figure 6), it is anticipated that the accumulation of other apoplastic PR proteins is also reduced in the mutant. Loss or suppression of the above responses is typically associated with enhanced susceptibility to pathogens [61,62]. For example, mutation of *PEROXIDASE 33* (*PRX33*; At3g49110) and *PRX34* (At3g49120) reduced ROS production and callose deposition in response to MAMPs and increased susceptibility to virulent *Pst* [63], while mutation of the callose synthase gene *CalS12* conferred susceptibility to *Pst hrcC* ^*-*^ [43]. Furthermore, several *P. syringae* T3SEs effectively block MTI and facilitate pathogen growth by suppressing apoplastic defence responses. These include HopN1, which suppresses the production of ROS [64], AvrPto, which suppresses callose deposition [43,47,51], and HopM1, AvrRpt2 and AvrRpm1 which suppress callose deposition and PR protein accumulation [43,51]. Indeed, analysis of apoplastic responses in the *tga1-1 tga4-1* mutant required the use of bacterial strains, including the T3SS-deficient mutant *Pst hrcC* ^*-*^ and the nonhost pathogen *Psp*, that have limited capacity to suppress *Arabidopsis* innate defences. Thus, it is reasonable to postulate that increased susceptibility of the *tga1-1 tga4-1* mutant to these bacteria (Figure 1) is attributed, at least in part, to impaired apoplastic defences.

The use of *Pst hrcC* ^*-*^ and *Psp*, as well as a purified MAMP, also allowed us to elucidate more directly the requirement of clade I TGA factors for MTI. Our results (Figure 1) indicate that these transcription factors are positive regulators of MTI and act in an NPR1-independent manner. The cofactor NPR1, which interacts with clade I TGA factors following SA elicitation [38], is a key regulator of SAR and disease resistance against virulent biotrophic pathogen (i.e. ETS) [41]. However, *npr1* mutants are not affected in MAMP-induced resistance against virulent *P. syringae* [28,65] and basal resistance against *Pst hrcC* ^*-*^ or *Psp* (Figure 1) [50,51], suggesting a limited role of NPR1 in MTI-mediated disease resistance. Furthermore, previous research was able to reveal a role for NPR1 in resistance against *Psp* by combining the *npr1* mutation with *pmr4*, which is defective in *CalS12*, and infecting with a strain harboring the T3SE HopM1 [51]. The finding that the *npr1-1 tga1-1 tga4-1* triple mutant is no more susceptible to *Pst hrcC* ^*-*^ or *Psp* than the *tga1-1 tga4-1* double mutant (Figure 1) failed to provide evidence for additive or synergistic genetic interactions between clade I TGA factors and NPR1 towards MTI. The requirement for HopM1 was not tested.

The observation that steady-state levels of defence gene transcripts, in particular *CalS12* (Figure S2) and *PR-1* (Figure 2A), are induced in *tga1-1 tga4-1* plants suggests that the mutant is compromised in some aspect of posttranscriptional regulation required for the induction of apoplastic defence responses. Such responses necessitate massive increases in protein secretion to accommodate the *de novo* synthesis of PR proteins, many of which are synthesized with an N-terminal signal peptide determining translocation into the ER, followed by secretion into the apoplast [16]. Analysis of mutants in components and regulators of the ER folding and secretory machinery has confirmed that failure to adapt to the increased demand for protein secretion leads to reduction of apoplastic PR-1 and impairs resistance against pathogens [24,25,28]. Similarly, callose deposition in papillae is delayed in mutants defective in vesicle-associated secretion processes [66], implicating callose precursors and/or the callose synthase protein as a defence component delivered to infection sites by the vesicle-related secretion pathway. At least one T3SE, HopM1, suppresses MTI responses by interfering with vesicle trafficking [67]. Moreover, silencing of a gene implicated in vesicle trafficking, the *Nicotiana* *benthamiana* syntaxin NbSYP132, resulted in a delay of PR accumulation in the cell wall after inoculation with *P. syringae* pv. *tabacina* [68].

The antibiotic TM specifically inhibits the synthesis of *N*-linked glycans in *Arabidopsis* [26] and is widely used to trigger ER-stress and the subsequent UPR in plants [15], although the accumulation of unfolded protein following treatment often is not confirmed. Increased sensitivity of *tga1-1 tga4-1* seedlings to TM (Figure 6) and associated upregulation of ER-resident genes encoding chaperones (Figure 7) suggest that loss of clade I TGA factors impairs ER-based protein folding and/or secretion, which may account for the observed defects in apoplastic defence responses. Of note, mutants in several components of the ER secretion system are also overly sensitive to TM. These include ER-resident chaperones and co-chaperones such as BiP2 [24] and BCL-2-ASSOCIATED ATHANOGENE 7 (BAG7) [69], enzymes involved in protein *N*-glycosylation such as LEAF WILTING 1, a *cis*-prenyltransferase required for dolichol biosynthesis [70], and regulators of the UPR such as IRE1 [25,71] and GTP-binding protein β1 (AGB1), a G protein subunit [71]. Mutation of TBF1 that regulates the expression of several genes involved in ER-based protein folding and secretion in response to elf18 and SA also confers TM sensitivity [28].

A characteristic of the UPR in plants is the upregulation of genes encoding ER-resident proteins involved in protein folding and degradation to bring capacity in line with demand [15]. The *Arabidopsis* UPR sensors bZIP28 (At3g10800), bZIP60 and IRE1 are important positive regulators of UPR gene expression, including *BiP*_*1/2*_ and *BiP3*, and their loss impairs TM-induced UPR marker gene expression [71-73]. In contrast, *tga1-1 tga4-1* seedlings continue to accumulate *BiP*_*1/2*_ and *BiP3* following treatment with TM (Figure 7), indicating that their increased sensitivity to this antibiotic is not due to the loss of ER-resident chaperone gene expression. Under ER stress, the IRE1 ribonuclease catalyzes the splicing of *bZIP60* in the cytoplasm to generate an mRNA species capable of encoding a functional, nuclear localized transcription factor [74,75]. This splicing event occurs in *tga1-1 tga4-1* seedlings after treatment with TM (Figure S5), suggesting that loss of clade I TGA factors does not impair the IRE1/bZIP60 branch of UPR signaling, which is required for ETS and SAR against *P. syringae* [25].

During SAR, upregulation of ER related genes is dependent on NPR1 [24]. The NPR1-dependent genes encoding ER proteins are enriched in the *TL1 cis*-element [24] that is recognized by the heat shock factor-like protein TBF1, but not by TGA factors [24,28]. In addition, clade I TGA factors were not required for SAR and NPR1-dependent ER gene expression after SA treatment [32]. Conversely, NPR1 has a limited role in disease resistance against *Pst hrcC* ^*-*^ and *Psp*, which are compromised in *tga1-1 tga4-1* mutant plant (Figure 1) [50,51]. It has also been reported that induction of ER genes after treatment with a cell wall degrading enzyme, secreted by a bacterial pathogen, is SA- and NPR1-independent [76]. Based on these findings, we postulate that clade I TGA factors regulate an NPR1-independent ER secretion pathway during defence.

Indeed, *tga1-1 tga4-1* seedlings accumulate more transcripts of ER-chaperone genes than the wild type following TM treatment (Figure 7). Higher levels of *BiP* transcripts could be related to the greater ER stress experienced by the mutant (Figure 6). This is consistent with a report that *BiP3* expression increases as a function of TM concentration [26], and hence the severity of ER stress. Similar to *tga1-1 tga4-1*, the *bip2* and *bag7* mutants are hypersensitive to TM and express higher levels of UPR genes than wild type, although in response to ER stress induced by BTH [24] or heat and cold [69], respectively. Increased expression of the *BiP3* chaperone gene in the *bag7* mutant was speculated to be a compensation mechanism for the loss of the BAG7 co-chaperone [69]. Whether loss of clade I TGA factors compromises the expression of genes required to alleviate ER stress, other than the well-characterized ER-resident chaperones, remains to be demonstrated.

Sensitivity to TM coupled with upregulation of UPR genes by TM was reported for the *agb1-3* mutant [71]. It was proposed that AGB1 is a negative regulator of UPR gene expression, keeping transcript levels in check to prevent the induction of apoptosis. We have previously speculated that clade I TGA factors may act as negative regulators of SA-induced PR gene expression [32]. However, unlike promoters of these PR genes, those of genes involved in ER protein folding and secretion are not enriched for the consensus binding motif of TGA factors [24]. This suggests a more indirect role of clade I TGA factors in regulating BiP gene expression, and accordingly, we favor a model whereby increased levels of ER-resident chaperone gene expression is attributed to greater stress or compensation for loss of other, currently unknown, UPR effectors.

The observation that levels of both total and apoplastic PR-1 are reduced in the *tga1-1 tga4-1* mutant (Figure 5), but only apoplastic PR-1 is impaired in *npr1*, *tbf1* and *ire1* mutants [24,25,28] may indicate an additional role for clade I TGA factors in regulating the translation or degradation of ER proteins, both known control points of the UPR [15]. Alternatively, the differences in results could be attributed to the use of distinct ER stress inducer between studies: the current research analyzed PR-1 levels following challenge with *Pst hrcC* ^*-*^ and *Psp* while others treated with SA or BTH.

Recent studies have highlighted the differential role of various components of the ER folding and secretion machinery in regulating plant innate resistance. For example, mutation of several genes in the *N*-glycosylation pathway impair EFR biogenesis and elf18-mediated responses with minimal effects on FLS2/flg22 responses [19-22]. Activation of plant defence responses is energetically demanding and treatment of plants with purified MAMPs inhibits growth [2]. Impairment of PRR biogenesis due to mutation of components of the ER protein secretion machinery renders plants less sensitive to the growth inhibitory effects of purified MAMPs. However, the *tga1-1 tga4-1* seedlings continue to display sensitivity to elf18 and flg22 (Figure S4). This suggests that clade I TGA factors do not regulate the secretion pathways responsible for EFR and FLS2 biogenesis but those modulating downstream events.

## Supporting Information

Figure S1 **Growth of *Pst hrcC*^*-*^ and *Psp* 1448a in Col-0, *tga1-1*, *tga4-1* and *tga1-1 tga4-1* mutant plants.** Four-week-old leaves were syringe-infiltrated with a bacterial suspension (*Pst* *hrcC* ^*-*^ at 10^5^ colony forming units (cfu) ml^-1^ [A] or *Psp* 1448a at 10^6^ cfu ml^-1^ [B]). Bacterial titres were measured at 4 days after inoculation. The error bars represent the standard deviation of six replicates. An ANOVA of the log-transformed data was performed at α = 0.05; treatments with common letters over bars are not significantly different from each other. Post-hoc tests are presented in Table S2.
(TIF)

Figure S2 **Callose synthase gene expression in Col-0 and the *tga1-1 tga4-1* plants.** Four-week-old leaves were syringe-infiltrated with 10^8^ cfu ml^-1^ of *Pst* *hrcC* ^*-*^. Leaf tissues from three plants were collected and pooled as one sample for RNA isolation. Values were normalized to the expression of *UBIQUITIN5*. The error bars represent the standard deviation of three biological samples. Student’s *t*-tests were performed between Col-0 and *tga1-1* *tga4-1* at each time point (p<0.05).
(TIF)

Figure S3 **ER resident gene expression in Col-0 and the *tga1-1 tga4-1* plants.** Four-week-old leaves without any treatment were collected for RNA isolation. Leaf tissues from three plants were collected and pooled as one sample for RNA isolation. Values were normalized to the expression of *UBIQUITIN5*. The error bars represent the standard deviation of three biological samples. Student’s *t*-tests were performed between Col-0 and *tga1-1* *tga4-1* for each gene (p<0.05).
(TIF)

Figure S4 **MAMP-induced seedling growth inhibition in Col-0 and the *tga1-1 tga4-1* plants.** Five-day-old seedlings were transferred to liquid MS medium containing 1% sucrose supplemented with the indicated concentrations of peptides. BT indicates fresh weight of seedling before treatment. Fresh weight of seedlings was measured one week after treatment. Two seedlings were counted as one sample for measurement and 6 samples were measured for each genotype. Results are means ± standard deviation (n=6). Student’s *t*-tests were performed between Col-0 and the double mutant at each concentration (p<0.05) and none of the comparisons were found to be statistically significant. These experiments were repeated three times with similar results.
(TIF)

Figure S5 **Tunicamycin-activated *bZIP60* mRNA splicing in Col-0 and the *tga1-1 tga4-1* plants.** RNA samples were isolated from 10-day-old seedlings immersed with 5 µg ml^-1^ TM for the indicated time periods. **A**, RT-qPCR of *bZIP60* in Col-0 and the *tga1-1* *tga4-1* mutant after TM treatment. Values were normalized to the expression of *UBIQUITIN5*. The error bars represent the average ± standard deviation of four biological samples. **B**, Schematic representation of primer locations used for detection of unspliced or spliced *bZIP60* mRNA. Primers (bZIP60-P1/bZIP60-P2) are designed to detect both unspliced and spliced *bZIP60* mRNA. Primers (bZIP60-P3/bZIP60-P4) are designed to specifically detect spliced *bZIP60* mRNA. **C**, Detection of *bZIP60u* and *bZIP60s* cDNA after TM treatment. RT-qPCR was performed using the primer set bZIP60-P1/bZIP60-P2. **D**, Detection of *bZIP60s* cDNA after TM treatment. RT-PCR was performed using the primer set bZIP60-P3/bZIP60-P4.
(TIF)

Figure S6 **Extracellular PR-1 protein accumulation in Col-0 and *tga1-1 tga4-1* after *Pst hrcC*^*-*^ inoculation.** Four-week-old leaves were syringe-infiltrated with 10^8^ cfu ml^-1^ of *Pst* *hrcC* ^*-*^. Intercellular washing fluids (IWFs) were collected at indicated time point, separated on 16% Tricine-SDS-polyacrylamide gels and blotted with a PR-1 antibody. Replicates from three independent experiments were presented.
(TIF)

Table S1 **PCR oligonucleotides for RT-qPCR.**
(DOC)

Table S2 **Statistical analyses for disease test data presented in Figure 1 and Figure S1.**
(XLSX)

## References

[1] Jones JD, Dangl JL (2006) The plant immune system. Nature 444: 323-329. doi:10.1038/nature05286. PubMed: 17108957. 17108957 10.1038/nature05286

[2] Boller T, Felix G (2009) A renaissance of elicitors: perception of microbe-associated molecular patterns and danger signals by pattern-recognition receptors. Annu Rev Plant Biol 60: 379-406. doi:10.1146/annurev.arplant.57.032905.105346. PubMed: 19400727. 19400727 10.1146/annurev.arplant.57.032905.105346

[3] Boller T, He SY (2009) Innate immunity in plants: an arms race between pattern recognition receptors in plants and effectors in microbial pathogens. Science 324: 742-744. doi:10.1126/science.1171647. PubMed: 19423812. 19423812 10.1126/science.1171647 PMC2729760

[4] Durrant WE, Dong X (2004) Systemic acquired resistance. Annu Rev Phytopathol 42: 185-209. doi:10.1146/annurev.phyto.42.040803.140421. PubMed: 15283665. 15283665 10.1146/annurev.phyto.42.040803.140421

[5] Dempsey DA, Klessig DF (2012) SOS – too many signals for systemic acquired resistance? Trends Plant Sci 17: 538-545. doi:10.1016/j.tplants.2012.05.011. PubMed: 22749315. 22749315 10.1016/j.tplants.2012.05.011

[6] Vlot AC, Dempsey DA, Klessig DF (2009) Salicylic Acid, a multifaceted hormone to combat disease. Annu Rev Phytopathol 47: 177-206. doi:10.1146/annurev.phyto.050908.135202. PubMed: 19400653. 19400653 10.1146/annurev.phyto.050908.135202

[7] Dong X (2004) NPR1, all things considered. Curr Opin Plant Biol 7: 547-552. doi:10.1016/j.pbi.2004.07.005. PubMed: 15337097. 15337097 10.1016/j.pbi.2004.07.005

[8] Fu ZQ, Yan S, Saleh A, Wang W, Ruble J et al. (2012) NPR3 and NPR4 are receptors for the immune signal salicylic acid in plants. Nature 486: 228-232. PubMed: 22699612. 22699612 10.1038/nature11162 PMC3376392

[9] Wu Y, Zhang D, Chu JY, Boyle P, Wang Y et al. (2012) The Arabidopsis NPR1 protein is a receptor for the plant defense hormone salicylic acid. Cell Rep 1: 639-647. doi:10.1016/j.celrep.2012.05.008. PubMed: 22813739. 22813739 10.1016/j.celrep.2012.05.008

[10] Kwon C, Bednarek P, Schulze-Lefert P (2008) Secretory pathways in plant immune responses. Plant Physiol 147: 1575-1583. doi:10.1104/pp.108.121566. PubMed: 18678749. 18678749 10.1104/pp.108.121566 PMC2492620

[11] Tsuda K, Sato M, Glazebrook J, Cohen JD, Katagiri F (2008) Interplay between MAMP-triggered and SA-mediated defense responses. Plant J 53: 763-775. doi:10.1111/j.1365-313X.2007.03369.x. PubMed: 18005228. 18005228 10.1111/j.1365-313X.2007.03369.x

[12] Torres MA, Jones JD, Dangl JL (2006) Reactive oxygen species signaling in response to pathogens. Plant Physiol 141: 373-378. doi:10.1104/pp.106.079467. PubMed: 16760490. 16760490 10.1104/pp.106.079467 PMC1475467

[13] Hématy K, Cherk C, Somerville S (2009) Host-pathogen warfare at the plant cell wall. Curr Opin Plant Biol 12: 406-413. doi:10.1016/j.pbi.2009.06.007. PubMed: 19616468. 19616468 10.1016/j.pbi.2009.06.007

[14] van Loon LC, Rep M, Pieterse CM (2006) Significance of inducible defense-related proteins in infected plants. Annu Rev Phytopathol 44: 135-162. doi:10.1146/annurev.phyto.44.070505.143425. PubMed: 16602946. 16602946 10.1146/annurev.phyto.44.070505.143425

[15] Liu JX, Howell SH (2010) Endoplasmic reticulum protein quality control and its relationship to environmental stress responses in plants. Plant Cell 22: 2930-2942. doi:10.1105/tpc.110.078154. PubMed: 20876830. 20876830 10.1105/tpc.110.078154 PMC2965551

[16] Wang D, Dong X (2011) A highway for war and peace: the secretory pathway in plant-microbe interactions. Mol Plants 4: 581-587. doi:10.1093/mp/ssr053. 10.1093/mp/ssr053 PMC3146739 21742620

[17] Schröder M, Kaufman RJ (2005) The mammalian unfolded protein response. Annu Rev Biochem 74: 739-789. doi:10.1146/annurev.biochem.73.011303.074134. PubMed: 15952902. 15952902 10.1146/annurev.biochem.73.011303.074134

[18] Rutkowski DT, Kaufman RJ (2004) A trip to the ER: coping with stress. Trends Cell Biol 14: 20-28. doi:10.1016/j.tcb.2003.11.001. PubMed: 14729177. 14729177 10.1016/j.tcb.2003.11.001

[19] Li J, Zhao-Hui C, Batoux M, Nekrasov V, Roux M et al. (2009) Specific ER quality control components required for biogenesis of the plant innate immune receptor EFR. Proc Natl Acad Sci U S A 106: 15973-15978. doi:10.1073/pnas.0905532106. PubMed: 19717464. 19717464 10.1073/pnas.0905532106 PMC2747228

[20] Lu X, Tintor N, Mentzel T, Kombrink E, Boller T et al. (2009) Uncoupling of sustained MAMP receptor signaling from early outputs in an Arabidopsis endoplasmic reticulum glucosidase II allele. Proc Natl Acad Sci U S A 106: 22522-22527. doi:10.1073/pnas.0907711106. PubMed: 20007779. 20007779 10.1073/pnas.0907711106 PMC2799704

[21] Nekrasov V, Li J, Batoux M, Roux M, Chu ZH et al. (2009) Control of the pattern-recognition receptor EFR by an ER protein complex in plant immunity. EMBO J 28: 3428-3438. doi:10.1038/emboj.2009.262. PubMed: 19763086. 19763086 10.1038/emboj.2009.262 PMC2776097

[22] Saijo Y, Tintor N, Lu X, Rauf P, Pajerowska-Mukhtar K et al. (2009) Receptor quality control in the endoplasmic reticulum for plant innate immunity. EMBO J 28: 3439-3449. doi:10.1038/emboj.2009.263. PubMed: 19763087. 19763087 10.1038/emboj.2009.263 PMC2776098

[23] Häweker H, Rips S, Koiwa H, Salomon S, Saijo Y, Chinchilla D, Robatzek S, von SA (2010) Pattern recognition receptors require N-glycosylation to mediate plant immunity. J Biol Chem 285: 4629-4636. doi:10.1074/jbc.M109.063073. PubMed: 20007973. 20007973 10.1074/jbc.M109.063073 PMC2836068

[24] Wang D, Weaver ND, Kesarwani M, Dong X (2005) Induction of protein secretory pathway is required for systemic acquired resistance. Science 308: 1036-1040. doi:10.1126/science.1108791. PubMed: 15890886. 15890886 10.1126/science.1108791

[25] Moreno AA, Mukhtar MS, Blanco F, Boatwright JL, Moreno I et al. (2012) IRE1/bZIP60-mediated unfolded protein response plays distinct roles in plant immunity and abiotic stress responses. PLOS ONE 7: e31944. doi:10.1371/journal.pone.0031944. PubMed: 22359644. 22359644 10.1371/journal.pone.0031944 PMC3281089

[26] Koizumi N, Ujino T, Sano H, Chrispeels MJ (1999) Overexpression of a gene that encodes the first enzyme in the biosynthesis of asparagine-linked glycans makes plants resistant to tunicamycin and obviates the tunicamycin-induced unfolded protein response. Plant Physiol 121: 353-361. doi:10.1104/pp.121.2.353. PubMed: 10517826. 10517826 10.1104/pp.121.2.353 PMC59397

[27] Iwata Y, Koizumi N (2012) Plant transducers of the endoplasmic reticulum unfolded protein response. Trends Plant Sci 17: 720-727. doi:10.1016/j.tplants.2012.06.014. PubMed: 22796463. 22796463 10.1016/j.tplants.2012.06.014

[28] Pajerowska-Mukhtar KM, Wang W, Tada Y, Oka N, Tucker CL et al. (2012) The HSF-like transcription factor TBF1 is a major molecular switch for plant growth-to-defense transition. Curr Biol 22: 103-112. doi:10.1016/j.cub.2011.12.015. PubMed: 22244999. 22244999 10.1016/j.cub.2011.12.015 PMC3298764

[29] Zhang Y, Fan W, Kinkema M, Li X, Dong X (1999) Interaction of NPR1 with basic leucine zipper protein transcription factors that bind sequences required for salicylic acid induction of the PR-1 gene. Proc Natl Acad Sci U S A 96: 6523-6528. doi:10.1073/pnas.96.11.6523. PubMed: 10339621. 10339621 10.1073/pnas.96.11.6523 PMC26915

[30] Després C, DeLong C, Glaze S, Liu E, Fobert PR (2000) The Arabidopsis NPR1/NIM1 protein enhances the DNA binding activity of a subgroup of the TGA family of bZIP transcription factors. Plant Cell 12: 279-290. doi:10.2307/3870928. PubMed: 10662863. 10662863 PMC139764

[31] Zhou JM, Trifa Y, Silva H, Pontier D, Lam E et al. (2000) NPR1 differentially interacts with members of the TGA/OBF family of transcription factors that bind an element of the PR-1 gene required for induction by salicylic acid. Mol Plant Microbe Interact 13: 191-202. doi:10.1094/MPMI.2000.13.2.191. PubMed: 10659709. 10659709 10.1094/MPMI.2000.13.2.191

[32] Shearer HL, Cheng YT, Wang L, Liu J, Boyle P et al. (2012) Arabidopsis clade I TGA transcription factors regulate plant defenses in an NPR1-independent fashion. Mol Plant Microbe Interact 25: 1459-1468. doi:10.1094/MPMI-09-11-0256. PubMed: 22876961. 22876961 10.1094/MPMI-09-11-0256

[33] Shearer HL, Wang L, DeLong C, Després C, Fobert PR (2009) NPR1 enhances the DNA binding activity of the *Arabidopsis* bZIP transcription factor TGA7. Botany 87: 561-570. doi:10.1139/B08-143.

[34] Rochon A, Boyle P, Wignes T, Fobert PR, Després C (2006) The coactivator function of Arabidopsis NPR1 requires the core of its BTB/POZ domain and the oxidation of C-terminal cysteines. Plant Cell 18: 3670-3685. doi:10.1105/tpc.106.046953. PubMed: 17172357. 17172357 10.1105/tpc.106.046953 PMC1785396

[35] Zhang Y, Tessaro MJ, Lassner M, Li X (2003) Knockout analysis of Arabidopsis transcription factors TGA2, TGA5, and TGA6 reveals their redundant and essential roles in systemic acquired resistance. Plant Cell 15: 2647-2653. doi:10.1105/tpc.014894. PubMed: 14576289. 14576289 10.1105/tpc.014894 PMC280568

[36] Kesarwani M, Yoo J, Dong X (2007) Genetic interactions of TGA transcription factors in the regulation of pathogenesis-related genes and disease resistance in Arabidopsis. Plant Physiol 144: 336-346. doi:10.1104/pp.106.095299. PubMed: 17369431. 17369431 10.1104/pp.106.095299 PMC1913812

[37] Choi J, Huh SU, Kojima M, Sakakibara H, Paek KH et al. (2010) The cytokinin-activated transcription factor ARR2 promotes plant immunity via TGA3/NPR1-dependent salicylic acid signaling in Arabidopsis. Dev Cell 19: 284-295. doi:10.1016/j.devcel.2010.07.011. PubMed: 20708590. 20708590 10.1016/j.devcel.2010.07.011

[38] Després C, Chubak C, Rochon A, Clark R, Bethune T et al. (2003) The Arabidopsis NPR1 disease resistance protein is a novel cofactor that confers redox regulation of DNA binding activity to the basic domain/leucine zipper transcription factor TGA1. Plant Cell 15: 2181-2191. doi:10.1105/tpc.012849. PubMed: 12953119. 12953119 10.1105/tpc.012849 PMC181339

[39] Lindermayr C, Sell S, Müller B, Leister D, Durner J (2010) Redox regulation of the NPR1-TGA1 system of Arabidopsis thaliana by nitric oxide. Plant Cell 22: 2894-2907. doi:10.1105/tpc.109.066464. PubMed: 20716698. 20716698 10.1105/tpc.109.066464 PMC2947166

[40] Hirano SS, Upper CD (1990) Population Biology and Epidemiology of Pseudomonas Syringae. Annu Rev Phytopathol 28: 155-177. doi:10.1146/annurev.phyto.28.1.155.

[41] Cao H, Bowling SA, Gordon AS, Dong X (1994) Characterization of an Arabidopsis Mutant That Is Nonresponsive to Inducers of Systemic Acquired Resistance. Plant Cell 6: 1583-1592. doi:10.2307/3869945. PubMed: 12244227. 12244227 10.1105/tpc.6.11.1583 PMC160545

[42] Armitage P (1971) Statistical Methods in Medical Research. Blackwell Scientific Publication.

[43] Kim MG, da CL, McFall AJ, Belkhadir Y, Debroy S et al. (2005) Two Pseudomonas syringae type III effectors inhibit RIN4-regulated basal defense in Arabidopsis. Cell 121: 749-759. doi:10.1016/j.cell.2005.03.025. PubMed: 15935761. 15935761 10.1016/j.cell.2005.03.025

[44] Gómez-Gómez L, Felix G, Boller T (1999) A single locus determines sensitivity to bacterial flagellin in Arabidopsis thaliana. Plant J 18: 277-284. doi:10.1046/j.1365-313X.1999.00451.x. PubMed: 10377993. 10377993 10.1046/j.1365-313x.1999.00451.x

[45] Schägger H (2006) Tricine-SDS-PAGE. Nat Protoc 1: 16-22. doi:10.1038/nprot.2006.4. PubMed: 17406207. 17406207 10.1038/nprot.2006.4

[46] Kliebenstein DJ, Dietrich RA, Martin AC, Last RL, Dangl JL (1999) LSD1 regulates salicylic acid induction of copper zinc superoxide dismutase in Arabidopsis thaliana. Mol Plant Microbe Interact 12: 1022-1026. doi:10.1094/MPMI.1999.12.11.1022. PubMed: 10550898. 10550898 10.1094/MPMI.1999.12.11.1022

[47] Hauck P, Thilmony R, He SY (2003) A Pseudomonas syringae type III effector suppresses cell wall-based extracellular defense in susceptible Arabidopsis plants. Proc Natl Acad Sci U S A 100: 8577-8582. doi:10.1073/pnas.1431173100. PubMed: 12817082. 12817082 10.1073/pnas.1431173100 PMC166271

[48] Mysore KS, Ryu CM (2004) Nonhost resistance: how much do we know? Trends Plant Sci 9: 97-104. doi:10.5363/tits.9.7_97. PubMed: 15102376. 15102376 10.1016/j.tplants.2003.12.005

[49] Forsyth A, Mansfield JW, Grabov N, de TM, Sinapidou E et al. (2010) Genetic dissection of basal resistance to Pseudomonas syringae pv. phaseolicola in accessions of Arabidopsis. Mol Plant Microbe Interact 23: 1545-1552. doi:10.1094/MPMI-02-10-0047. PubMed: 20653411. 20653411 10.1094/MPMI-02-10-0047

[50] van Wees SC, Glazebrook J (2003) Loss of non-host resistance of Arabidopsis NahG to Pseudomonas syringae pv. phaseolicola is due to degradation products of salicylic acid. Plant J 33: 733-742. doi:10.1046/j.1365-313X.2003.01665.x. PubMed: 12609045. 12609045 10.1046/j.1365-313x.2003.01665.x

[51] Ham JH, Kim MG, Lee SY, Mackey D (2007) Layered basal defenses underlie non-host resistance of Arabidopsis to Pseudomonas syringae pv. phaseolicola. Plant J 51: 604-616. doi:10.1111/j.1365-313X.2007.03165.x. PubMed: 17573803. 17573803 10.1111/j.1365-313X.2007.03165.x

[52] Rohde A, Morreel K, Ralph J, Goeminne G, Hostyn V et al. (2004) Molecular phenotyping of the pal1 and pal2 mutants of Arabidopsis thaliana reveals far-reaching consequences on phenylpropanoid, amino acid, and carbohydrate metabolism. Plant Cell 16: 2749-2771. doi:10.1105/tpc.104.023705. PubMed: 15377757. 15377757 10.1105/tpc.104.023705 PMC520969

[53] Mishina TE, Zeier J (2007) Bacterial non-host resistance: interactions of Arabidopsis with non-adapted Pseudomonas syringae strains. Physiol Plant 131: 448-461. doi:10.1111/j.1399-3054.2007.00977.x. PubMed: 18251883. 18251883 10.1111/j.1399-3054.2007.00977.x

[54] Asai T, Tena G, Plotnikova J, Willmann MR, Chiu WL et al. (2002) MAP kinase signalling cascade in Arabidopsis innate immunity. Nature 415: 977-983. doi:10.1038/415977a. PubMed: 11875555. 11875555 10.1038/415977a

[55] Lu M, Tang X, Zhou JM (2001) Arabidopsis NHO1 is required for general resistance against Pseudomonas bacteria. Plant Cell 13: 437-447. doi:10.2307/3871287. PubMed: 11226196. 11226196 10.1105/tpc.13.2.437 PMC102253

[56] Kang L, Li J, Zhao T, Xiao F, Tang X et al. (2003) Interplay of the Arabidopsis nonhost resistance gene NHO1 with bacterial virulence. Proc Natl Acad Sci U S A 100: 3519-3524. doi:10.1073/pnas.0637377100. PubMed: 12626746. 12626746 10.1073/pnas.0637377100 PMC152325

[57] Nicaise V, Roux M, Zipfel C (2009) Recent advances in PAMP-triggered immunity against bacteria: pattern recognition receptors watch over and raise the alarm. Plant Physiol 150: 1638-1647. doi:10.1104/pp.109.139709. PubMed: 19561123. 19561123 10.1104/pp.109.139709 PMC2719144

[58] Jacobs AK, Lipka V, Burton RA, Panstruga R, Strizhov N et al. (2003) An Arabidopsis Callose Synthase, GSL5, Is Required for Wound and Papillary Callose Formation. Plant Cell 15: 2503-2513. doi:10.1105/tpc.016097. PubMed: 14555698. 14555698 10.1105/tpc.016097 PMC280557

[59] Nishimura MT, Stein M, Hou BH, Vogel JP, Edwards H et al. (2003) Loss of a callose synthase results in salicylic acid-dependent disease resistance. Science 301: 969-972. doi:10.1126/science.1086716. PubMed: 12920300. 12920300 10.1126/science.1086716

[60] Dong X, Hong Z, Chatterjee J, Kim S, Verma DP (2008) Expression of callose synthase genes and its connection with Npr1 signaling pathway during pathogen infection. Planta 229: 87-98. doi:10.1007/s00425-008-0812-3. PubMed: 18807070. 18807070 10.1007/s00425-008-0812-3

[61] Hückelhoven R (2007) Cell wall-associated mechanisms of disease resistance and susceptibility. Annu Rev Phytopathol 45: 101-127. doi:10.1146/annurev.phyto.45.062806.094325. PubMed: 17352660. 17352660 10.1146/annurev.phyto.45.062806.094325

[62] Göhre V, Robatzek S (2008) Breaking the barriers: microbial effector molecules subvert plant immunity. Annu Rev Phytopathol 46: 189-215. doi:10.1146/annurev.phyto.46.120407.110050. PubMed: 18422429. 18422429 10.1146/annurev.phyto.46.120407.110050

[63] Daudi A, Cheng Z, O’Brien JA, Mammarella N, Khan S et al. (2012) The apoplastic oxidative burst peroxidase in Arabidopsis is a major component of pattern-triggered immunity. Plant Cell 24: 275-287. doi:10.1105/tpc.111.093039. PubMed: 22247251. 22247251 10.1105/tpc.111.093039 PMC3289579

[64] Rodríguez-Herva JJ, González-Melendi P, Cuartas-Lanza R, Antúnez-Lamas M, Río-Alvarez I et al. (2012) A bacterial cysteine protease effector protein interferes with photosynthesis to suppress plant innate immune responses. Cell Microbiol 14: 669-681. doi:10.1111/j.1462-5822.2012.01749.x. PubMed: 22233353. 22233353 10.1111/j.1462-5822.2012.01749.x

[65] Zipfel C, Robatzek S, Navarro L, Oakeley EJ, Jones JD et al. (2004) Bacterial disease resistance in Arabidopsis through flagellin perception. Nature 428: 764-767. doi:10.1038/nature02485. PubMed: 15085136. 15085136 10.1038/nature02485

[66] Kwon C, Neu C, Pajonk S, Yun HS, Lipka U et al. (2008) Co-option of a default secretory pathway for plant immune responses. Nature 451: 835-840. doi:10.1038/nature06545. PubMed: 18273019. 18273019 10.1038/nature06545

[67] Nomura K, Mecey C, Lee YN, Imboden LA, Chang JH et al. (2011) Effector-triggered immunity blocks pathogen degradation of an immunity-associated vesicle traffic regulator in Arabidopsis. Proc Natl Acad Sci U S A 108: 10774-10779. doi:10.1073/pnas.1103338108. PubMed: 21670267. 21670267 10.1073/pnas.1103338108 PMC3127868

[68] Kalde M, Nühse TS, Findlay K, Peck SC (2007) The syntaxin SYP132 contributes to plant resistance against bacteria and secretion of pathogenesis-related protein 1. Proc Natl Acad Sci U S A 104: 11850-11855. doi:10.1073/pnas.0701083104. PubMed: 17592123. 17592123 10.1073/pnas.0701083104 PMC1913864

[69] Williams B, Kabbage M, Britt R, Dickman MB (2010) AtBAG7, an Arabidopsis Bcl-2-associated athanogene, resides in the endoplasmic reticulum and is involved in the unfolded protein response. Proc Natl Acad Sci U S A 107: 6088-6093. doi:10.1073/pnas.0912670107. PubMed: 20231441. 20231441 10.1073/pnas.0912670107 PMC2851922

[70] Zhang H, Ohyama K, Boudet J, Chen Z, Yang J et al. (2008) Dolichol biosynthesis and its effects on the unfolded protein response and abiotic stress resistance in Arabidopsis. Plant Cell 20: 1879-1898. doi:10.1105/tpc.108.061150. PubMed: 18612099. 18612099 10.1105/tpc.108.061150 PMC2518237

[71] Chen Y, Brandizzi F (2012) AtIRE1A/AtIRE1B and AGB1 independently control two essential unfolded protein response pathways in Arabidopsis. Plant J 69: 266-277. doi:10.1111/j.1365-313X.2011.04788.x. PubMed: 21914012. 21914012 10.1111/j.1365-313X.2011.04788.x

[72] Liu JX, Srivastava R, Che P, Howell SH (2007) An endoplasmic reticulum stress response in Arabidopsis is mediated by proteolytic processing and nuclear relocation of a membrane-associated transcription factor, bZIP28. Plant Cell 19: 4111-4119. doi:10.1105/tpc.106.050021. PubMed: 18156219. 18156219 10.1105/tpc.106.050021 PMC2217655

[73] Iwata Y, Fedoroff NV, Koizumi N (2008) Arabidopsis bZIP60 is a proteolysis-activated transcription factor involved in the endoplasmic reticulum stress response. Plant Cell 20: 3107-3121. doi:10.1105/tpc.108.061002. PubMed: 19017746. 19017746 10.1105/tpc.108.061002 PMC2613661

[74] Deng Y, Humbert S, Liu JX, Srivastava R, Rothstein SJ, Howell SH (2011) Heat induces the splicing by IRE1 of a mRNA encoding a transcription factor involved in the unfolded protein response in Arabidopsis. Proc Natl Acad Sci U S A 108: 7247-7252. doi:10.1073/pnas.1102117108. PubMed: 21482766. 21482766 10.1073/pnas.1102117108 PMC3084119

[75] Nagashima Y, Mishiba K, Suzuki E, Shimada Y, Iwata Y, Koizumi N (2011) Arabidopsis IRE1 catalyses unconventional splicing of bZIP60 mRNA to produce the active transcription factor. Sci Rep 1: 29 PubMed: 22355548. 22355548 10.1038/srep00029 PMC3216516

[76] Jelitto-Van Dooren EP, Vidal S, Denecke J (1999) Anticipating endoplasmic reticulum stress. A novel early response before pathogenesis-related gene induction. Plant Cell 11: 1935-1944. doi:10.1105/tpc.11.10.1935. PubMed: 10521523. 10521523 10.1105/tpc.11.10.1935 PMC144106

