# Supplementary figures and images for: Arabidopsis Clade I TGA Factors Regulate Apoplastic Defences against the Bacterial Pathogen Pseudomonas syringae through Endoplasmic Reticulum-Based Processes
Source: PLoS One. 2013 Sep 27;8(9):e77378. doi: 10.1371/journal.pone.0077378 (PMC3785447; doi:10.1371/journal.pone.0077378)

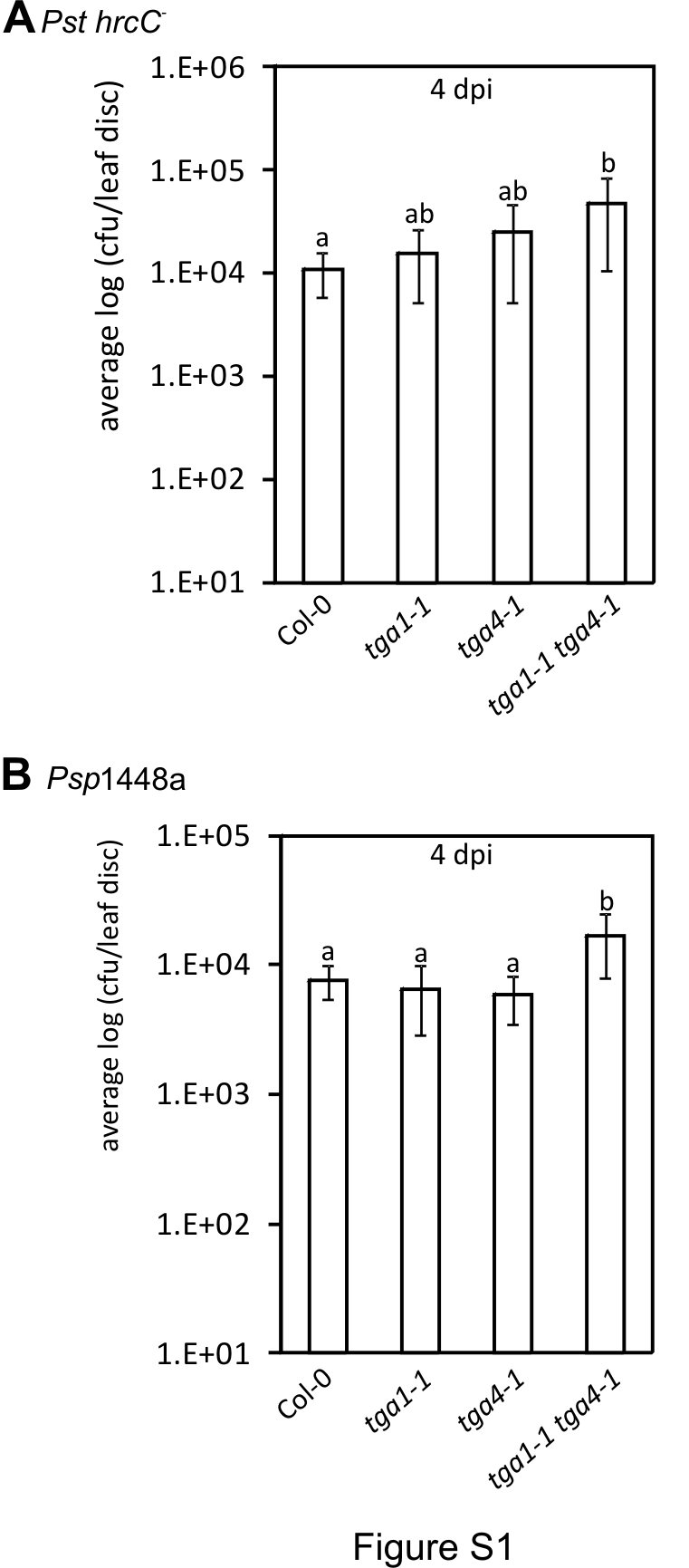

Supplement: Figure S1 — Growth of Pst hrcC- and Psp 1448a in Col-0, tga1-1, tga4-1 and tga1-1 tga4-1 mutant plants. Four-week-old leaves were syringe-infiltrated with a bacterial suspension (Pst hrcC - at 105 colony forming units (cfu) ml-1 [A] or Psp 1448a at 106 cfu ml-1 [B]). Bacterial titres were measured at 4 days after inoculation. The error bars represent the standard deviation of six replicates. An ANOVA of the log-transformed data was performed at α = 0.05; treatments with common letters over bars are not significantly different from each other. Post-hoc tests are presented in Table S2. (TIF) [file pone.0077378.s001.tif]

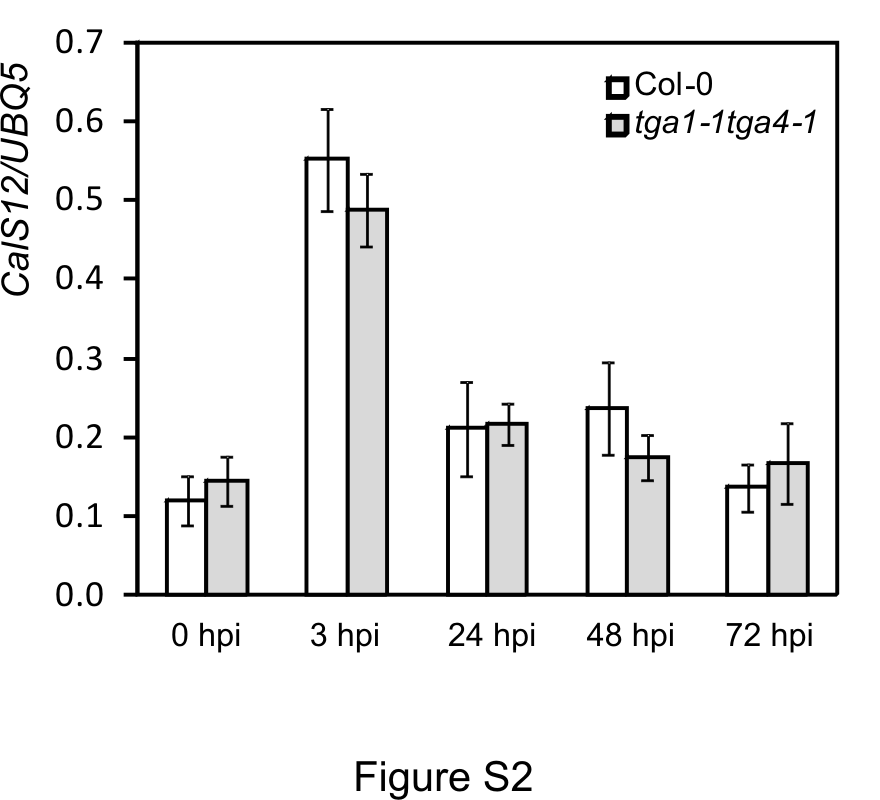

Supplement: Figure S2 — Callose synthase gene expression in Col-0 and the tga1-1 tga4-1 plants. Four-week-old leaves were syringe-infiltrated with 108 cfu ml-1 of Pst hrcC -. Leaf tissues from three plants were collected and pooled as one sample for RNA isolation. Values were normalized to the expression of UBIQUITIN5. The error bars represent the standard deviation of three biological samples. Student’s t-tests were performed between Col-0 and tga1-1 tga4-1 at each time point (p<0.05). (TIF) [file pone.0077378.s002.tif]

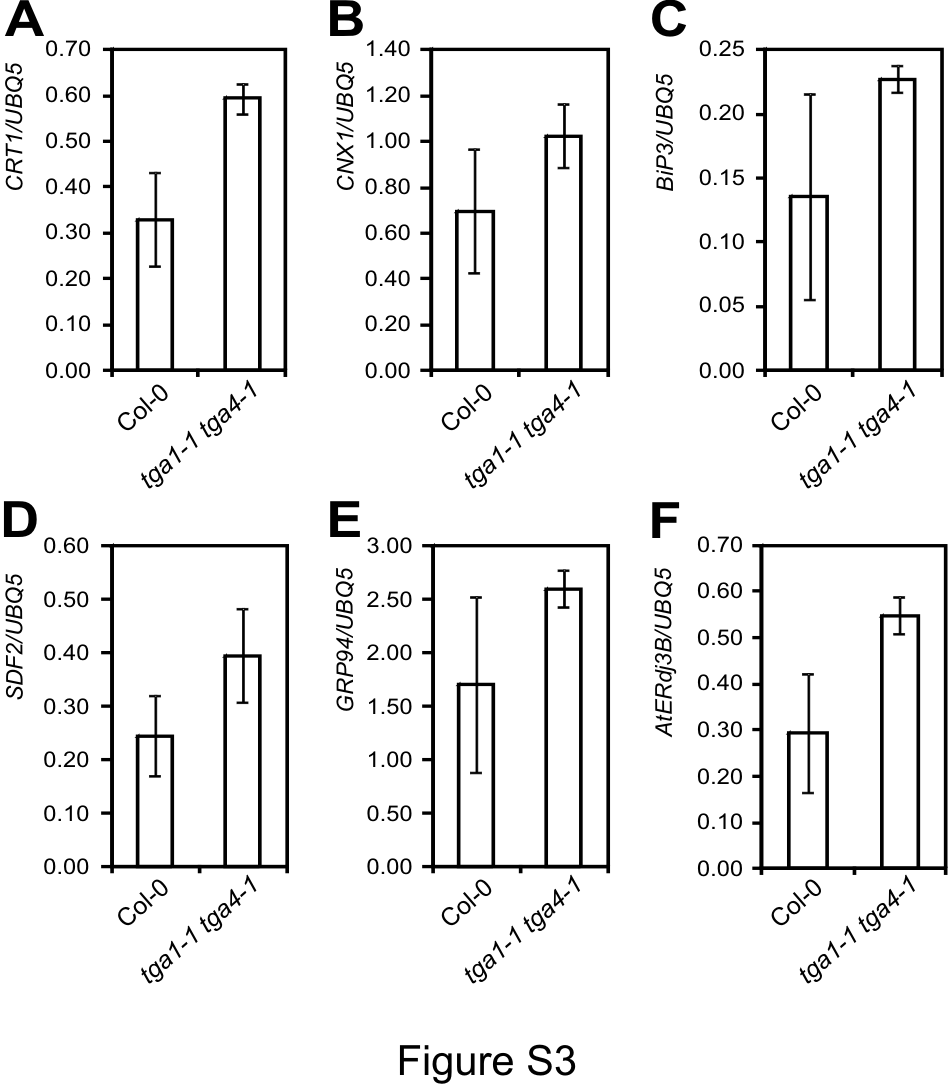

Supplement: Figure S3 — ER resident gene expression in Col-0 and the tga1-1 tga4-1 plants. Four-week-old leaves without any treatment were collected for RNA isolation. Leaf tissues from three plants were collected and pooled as one sample for RNA isolation. Values were normalized to the expression of UBIQUITIN5. The error bars represent the standard deviation of three biological samples. Student’s t-tests were performed between Col-0 and tga1-1 tga4-1 for each gene (p<0.05). (TIF) [file pone.0077378.s003.tif]

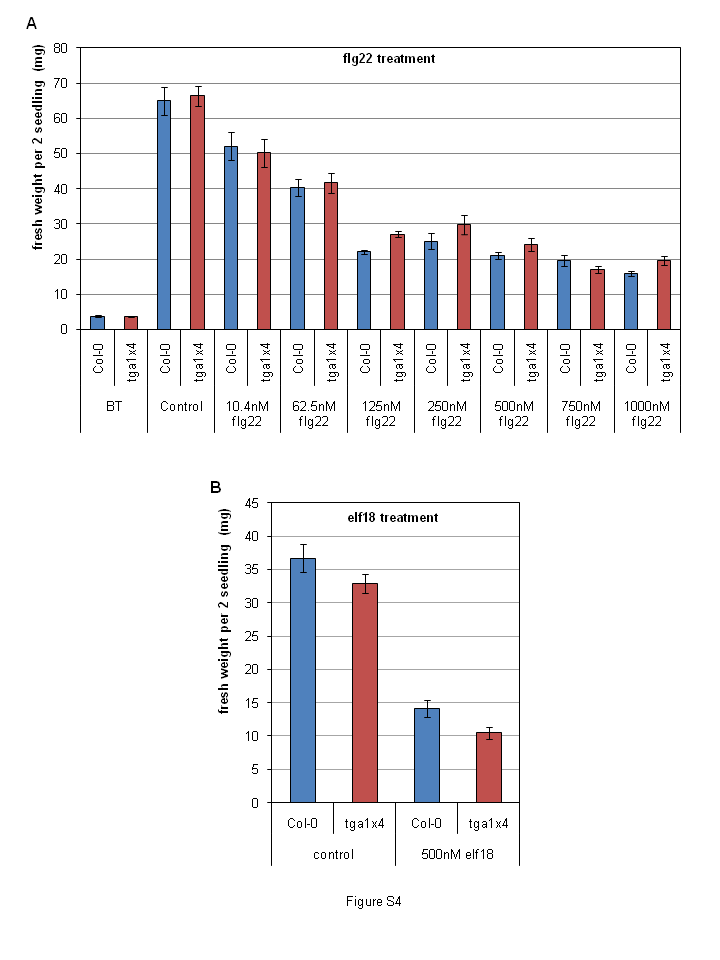

Supplement: Figure S4 — MAMP-induced seedling growth inhibition in Col-0 and the tga1-1 tga4-1 plants. Five-day-old seedlings were transferred to liquid MS medium containing 1% sucrose supplemented with the indicated concentrations of peptides. BT indicates fresh weight of seedling before treatment. Fresh weight of seedlings was measured one week after treatment. Two seedlings were counted as one sample for measurement and 6 samples were measured for each genotype. Results are means ± standard deviation (n=6). Student’s t-tests were performed between Col-0 and the double mutant at each concentration (p<0.05) and none of the comparisons were found to be statistically significant. These experiments were repeated three times with similar results. (TIF) [file pone.0077378.s004.tif]

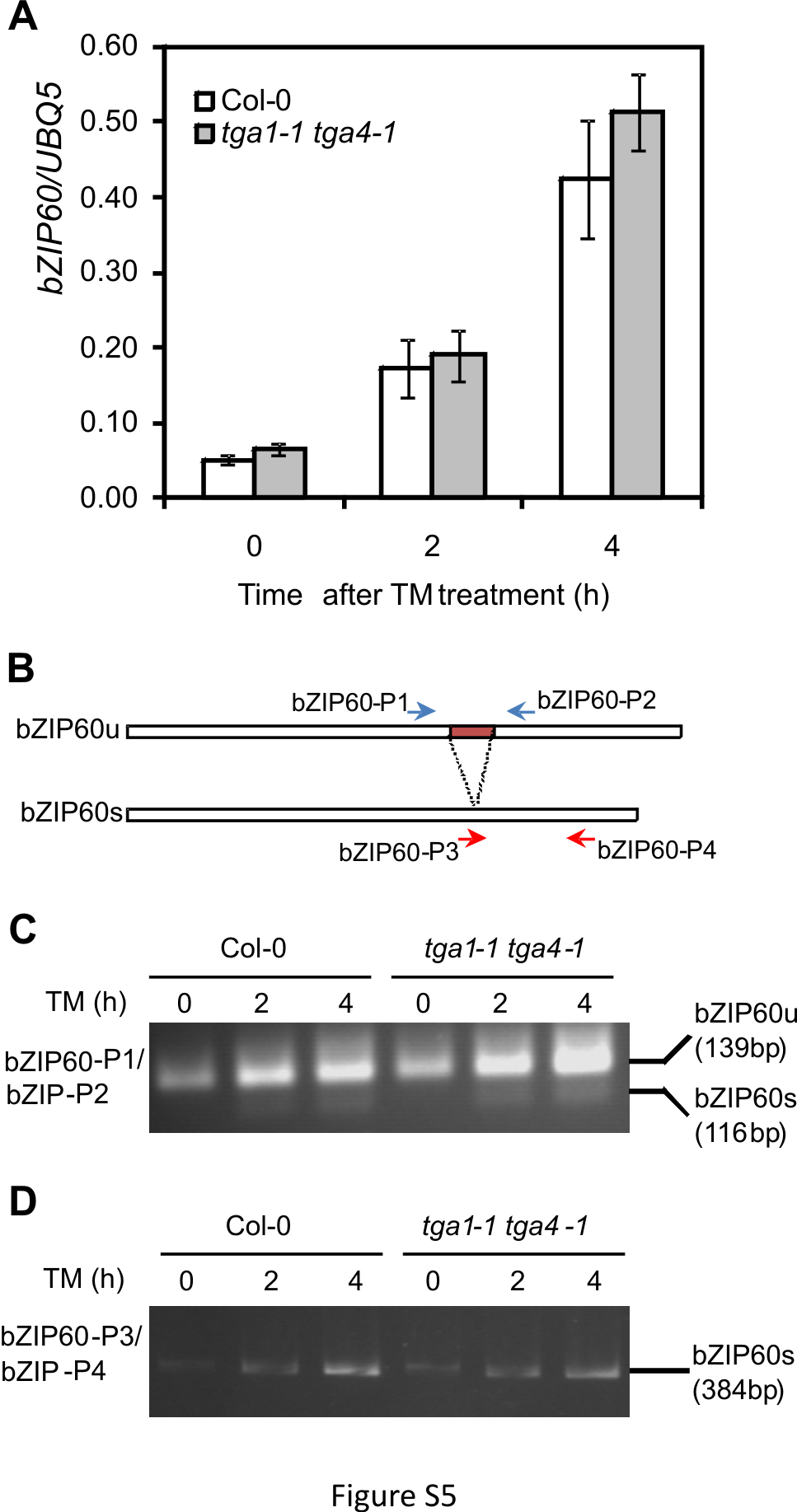

Supplement: Figure S5 — Tunicamycin-activated bZIP60 mRNA splicing in Col-0 and the tga1-1 tga4-1 plants. RNA samples were isolated from 10-day-old seedlings immersed with 5 µg ml-1 TM for the indicated time periods. A, RT-qPCR of bZIP60 in Col-0 and the tga1-1 tga4-1 mutant after TM treatment. Values were normalized to the expression of UBIQUITIN5. The error bars represent the average ± standard deviation of four biological samples. B, Schematic representation of primer locations used for detection of unspliced or spliced bZIP60 mRNA. Primers (bZIP60-P1/bZIP60-P2) are designed to detect both unspliced and spliced bZIP60 mRNA. Primers (bZIP60-P3/bZIP60-P4) are designed to specifically detect spliced bZIP60 mRNA. C, Detection of bZIP60u and bZIP60s cDNA after TM treatment. RT-qPCR was performed using the primer set bZIP60-P1/bZIP60-P2. D, Detection of bZIP60s cDNA after TM treatment. RT-PCR was performed using the primer set bZIP60-P3/bZIP60-P4. (TIF) [file pone.0077378.s005.tif]
