# Supplementary material for: Arabidopsis Clade I TGA Factors Regulate Apoplastic Defences against the Bacterial Pathogen Pseudomonas syringae through Endoplasmic Reticulum-Based Processes
Source: PLoS One. 2013 Sep 27;8(9):e77378. doi: 10.1371/journal.pone.0077378 (PMC3785447; doi:10.1371/journal.pone.0077378)
Supplement: Table S1 — PCR oligonucleotides for RT-qPCR. (DOC) [file pone.0077378.s007.doc]

Table S1 PCR oligonucleotides for k-RT-PCR

| Target | Primer name | 5’ to 3’ sequence | Efficiency (%) | Amplicon Size (bp) | T_m_ (˚C) |
| --- | --- | --- | --- | --- | --- |
| Oligos for Figure 2 | | | | | |
| PR1 At2g14610 | PR1F | GCTCTTGTAGGTGCTCTTGTTCTTCC | 104.4 | 173 | 66 |
|  | PR1R | AGTCTGCAGTTGCCTCTTAGTTGTTC |  |  |  |
| FRK1 At2g19190 | FRK1-Q5’ | ATTAGATGCAGCGCAAGGACTAGAGTATCTT | 101.6 | 124 | 62 |
|  | FRK1-Q3’ | GAAGTCCGCCATCTTCGCTTGGAGCTTCTC |  |  |  |
| NHO1 At1g80460 | NHO1-Q5' | CTTCTGATGCAGATTCAGGCTGATCTGATGG | 101.6 | 95 | 66 |
|  | NHO1-Q3' | CCAGCTGCATAGGCTGCTCCTAATGCTGTTGT |  |  |  |
| PAL1 At2g37040 | PAL1-Q5’ | GAGCTGCAGCGGAGCAAATGAAAGGTAGCC | 97.7 | 137 | 66 |
|  | PAL1-Q3’ | ACCAATAGTTGAGATCGCAGCCACTTGTCC |  |  |  |
| Oligos for Figure 7 | | | | | |
| BiP2 At5g42020 | At5g42020-Q5′ | AAGATGAGTCTCACGATGAGCTCTAGATGT | 97.7 | 211 | 62 |
|  | At5g42020-Q3′ | GAAGTAAAGTTATCAGATGAACATAAAGAG |  |  |  |
| BiP3 At1g09080 | At1g09080-Q5′ | AAGACGTCACAATCGATTACAATAACGAAC | 115.1 | 158 | 66 |
|  | At1g09080-Q3′ | TTGTACACATACGTTTCAAGCTTGTTCCTC |  |  |  |
| ERdj3B At3g62600 | At3g62600-Q5′ | GTTCCGAATCAGAACTGCACCACATGCCCG | 110.2 | 146 | 66 |
|  | At3g62600-Q3′ | AACTGATGTCAACTTCGTGATCATCCAAGT |  |  |  |
| Oligos for Figure S1 | | | | | |
| CalS12 At4g03550 | CalS12-Q5’ | TGAAGAATTACACTATTTTCTGGAATGCTG | 107.2 | 199 | 62 |
|  | CalS12-Q3’ | GATTTCTTCCCAGTGACAATCTGCATGATG |  |  |  |
| Oligos for Figure S2 | | | | | |
| CRT1 At1g56340 | At1g56340-Q5' | AAACCAAGGAAGCCGAGGAAACCGATGCCG | 102.4 | 211 | 62 |
|  | At1g56340-Q3' | AGGATTTTCTCAAAGTCTAAAACAAAGTTA |  |  |  |
| CNX1 At5g61790 | At5g61790-Q5’ | GGCAACCGAGACGTGATAATTAGAACAAGA | 100.8 | 151 | 66 |
|  | At5g61790-Q3’ | ATTTCTCAAACTTGGTGTTACACCATTAT |  |  |  |
| BiP3 At1g09080 | At1g09080-Q5′ | AAGACGTCACAATCGATTACAATAACGAAC | 115.1 | 158 | 66 |
|  | At1g09080-Q3′ | TTGTACACATACGTTTCAAGCTTGTTCCTC |  |  |  |
| SDF2 At2g25110 | At2g25110-Q5’ | GCGGCGAAGAAACCGAGTCCGGAATACCCA | 96.7 | 144 | 66 |
|  | At2g25110-Q3’ | GGCAGACGTGTAACCGGAATCAGGATCGAG |  |  |  |
| GRP94 At4g24190 | At4g24190-Q5’ | TTGGCTGGTGGTCTAAACATTGAAGCCGAA | 103.7 | 143 | 62 |
|  | At4g24190-Q3’ | TATTCGAGTAAAACGATGTTCTGCTTTAAA |  |  |  |
| ERdj3B At3g62600 | At3g62600-Q5′ | GTTCCGAATCAGAACTGCACCACATGCCCG | 110.2 | 146 | 66 |
|  | At3g62600-Q3′ | AACTGATGTCAACTTCGTGATCATCCAAGT |  |  |  |
| Oligos for Figure S4 | | | | | |
| bZIP60 At1g42990 | bZIP60-Q5’ | AAGAAATAGAGATGCGGCGGTTAGATCGAG | 103.0 | 164 | 66 |
|  | bZIP60-Q3’ | CCTTTTGCAAACAGTAACGTAGAGACTGGT |  |  |  |
| Oligos for reference gene | | | | | |
| Ubiquitin 5 At3g62250 | Ubiquitin5 5′a | ACCTACGTTTACCAGAAAGAAGGAGTTGAA | 102.9 | 102 | 66 |
|  | Ubiquitin5 3′a | AGCTTACAAAATTCCCAAATAGAAATGCAG |  |  |  |
